# Risk factors of neonatal sepsis in India: A systematic review and meta-analysis

**DOI:** 10.1371/journal.pone.0215683

**Published:** 2019-04-25

**Authors:** Shruti Murthy, Myron Anthony Godinho, Vasudeva Guddattu, Leslie Edward Simon Lewis, N. Sreekumaran Nair

**Affiliations:** 1 Department of Statistics, Prasanna School of Public Health, Manipal Academy of Higher Education, Manipal, Karnataka, India; 2 WHO Collaborating Centre for eHealth, School of Public Health and Community Medicine, University of New South Wales, Sydney, New South Wales, Australia; 3 Department of Paediatrics, Neonatology Unit, Kasturba Medical College, Manipal Academy of Higher Education, Manipal, Karnataka, India; 4 Department of Medical Biometrics & Informatics (Biostatistics), Jawaharlal Institute of Postgraduate Medical Education and Research (JIPMER), Puducherry, India; University of Mississippi Medical Center, UNITED STATES

## Abstract

**Background:**

The incidence of neonatal sepsis in India is the highest in the world. Evidence regarding its risk factors can guide clinical practice and prevention strategies.

**Objective:**

To review, assess and synthesize the available literature from India on the risk factors of sepsis among neonates.

**Methodology:**

A systematic review was conducted. We searched PubMed, CINAHL, Scopus, Web of Science, Popline, IndMed, Indian Science Abstracts and Google Scholar from inception up to March 23, 2018 to identify observational analytical studies reporting on risk factors of laboratory-confirmed neonatal sepsis in India. Two authors independently screened studies (title, abstract and full-text stages), extracted data, and assessed quality. A random-effects meta-analysis was performed as substantial heterogeneity was anticipated. Subgroup and sensitivity analyses were additionally performed. Effect size in our review included odds ratio and standardized mean difference.

**Results:**

Fifteen studies were included from 11,009 records, of which nine were prospective in design. Birthweight and gestational age at delivery were the most frequently reported factors. On meta-analyses, it was found that male sex (OR: 1.3, 95% CI: 1.02, 1.68), out born neonates (OR: 5.5, 95% CI: 2.39, 12.49), need for artificial ventilation (OR: 5.61; 95% CI: 8.21, 41.18), gestational age <37 weeks (OR: 2.05; 95% CI:1.40, 2.99) and premature rupture of membranes (OR:11.14, 95% CI: 5.54, 22.38) emerged as risk factors for neonatal sepsis. Included studies scored lowest on exposure assessment and confounding adjustment, which limited comparability. Inadequacy and variation in definitions and methodology affected the quality of included studies and increased heterogeneity.

**Conclusions:**

Male neonates, outborn admissions, need for artificial ventilation, gestational age <37 weeks and premature rupture of membranes are risk factors for sepsis among neonates in India. Robustly designed and reported research is urgently needed to confirm the role of other risk factors of neonatal sepsis in India.

## Introduction

Sepsis is the second major cause of mortality among neonates, killing more than one million neonates annually.[1] Neonatal sepsis, pneumonia and meningitis together result in up to a quarter of all newborn deaths.[2] Globally, of the three million annual neonatal sepsis cases (2202/ 1,00,000 live births), India has the highest incidence of clinical sepsis (17,000/ 1,00,000 live births).[3] The case fatality rate of sepsis among neonates ranges between 25% to 65% in India.[4, 5] These rates are likely to be underestimated, and more accurate data is expected from the ‘Global Maternal and Neonatal Sepsis Initiative’.[6–8]

Neonatal sepsis includes septicaemia, pneumonia, meningitis, osteomyelitis, arthritis and urinary tract infections,[9] and does not yet have a consensus case definition, especially for Low- and Middle- Income Countries (LMICs).[10–13] Clinical features are non-specific and are inefficient for identifying neonates with early-onset sepsis (EOS).[14] Culture results take up to 48 hours; have been found to be positive in 25% to 45% of cases;[5] and run the risk of false-positive/ low-yield results after antenatal antibiotic exposure.[15] Moreover, culture-testing facilities are absent from most district hospitals in India.[5] In this scenario, the prediction and diagnosis of neonatal sepsis relies on culture-independent diagnostics and risk factor-based scoring systems.[16]

The application of a risk-factor based approach for guiding management decisions has been debated with relation to its cost-effectiveness.[17, 18] It has, however, been shown to be one of the highly effective approaches for reducing neonatal early-onset sepsis (EOS)-based mortality in High Income Countries (HICs).[2] It is recommended that in resource-limited settings with a high neonatal mortality rate, such as in India, a combination of risk factors and clinical signs should guide “intrapartum and neonatal management”.[2, 15, 19]

Previous systematic reviews on risk factors of neonatal sepsis have individually focused on gene association,[20] pneumonia,[21] meningitis,[22] and maternal factors in neonatal early-onset sepsis (EOS),[2] particularly of Group B Streptococcal (GBS) aetiology.[23, 24] Evidence from reviews of risk factors has been utilized globally to guide the development of management guidelines for neonatal sepsis, and it is similarly recommended that such evidence be used to inform guideline development for management decisions in India.[25, 26] Additionally, such evidence can aid in defining research priorities, and developing ‘integrated prevention strategies’.[2, 7] Finally, such evidence can aid the design (e.g. risk-factor based eligibility criteria) of intervention studies on neonatal sepsis.[15] However, evidence on risk factors in management guidelines on neonatal sepsis in India is informed by a few primary studies, most of which do not account for intrapartum antibiotic prophylaxis.[9, 25, 27]

For the above reasons, we intended to review, assess and synthesize the literature on all available risk factors of six systemic infections (under the umbrella of sepsis) among neonates in India. To the best of our knowledge, this is the first systematic review and meta-analysis to addressing both neonatal and maternal factors in the Indian context.

## Materials and methods

This review will be used to inform, a larger mixed-methods study addressing the burden of neonatal systemic infection in India. This systematic review and meta-analysis has been reported in accordance with the ‘Preferred Reporting Items for Systematic reviews and Meta-analysis (PRISMA) guidelines (see S1 PRISMA Checklist).[28] A protocol was developed for our review (see S1 File) and registered on the ‘International prospective register of systematic reviews PROSPERO’ (ID: PROSPERO 2017 CRD42017053721), which can be accessed on their website (http://www.crd.york.ac.uk/PROSPERO/display_record.php?ID=CRD42017053721).

### Searches

#### Information sources

Two authors **(**SM and MG) searched PubMed, CINAHL (EBSCOHost), Scopus (Elsevier), Web of Science (Clarivate Analytics), Popline, IndMed (MedKnow), Indian Science Abstracts and Google Scholar up to March 23, 2018. Studies on the first 10 pages of results from Google Scholar from the year 2000 onwards were screened. Additionally, reference lists of included studies and systematic reviews were screened for potentially relevant studies, though systematic reviews themselves were excluded. Additionally, researchers were contacted to identify further studies.

#### Search strategy

A comprehensive search strategy including all possible risk factors for neonatal sepsis in India was developed according to recommendations of the Centre for Reviews and Dissemination’s (CRD) ‘Guidance for undertaking reviews in healthcare’,[29] a literature review, and in consultation with subject experts and an information scientist. Free text and database specific subject headings were included. No time or language restrictions were applied. A search strategy was first developed for PubMed (see S1 File) and subsequently adapted for the other databases.

### Eligibility criteria

Studies were included in the systematic review if they

- were of observational analytical (cohort, case-control and analytical cross-sectional) design, reporting on two outcome groups: one with sepsis and one without sepsis. If the study design was unclear/ poorly reported, but the study reported data with a comparison group, we classified the study design as either “prospective” (data collected when neonate was in the neonatal unit) or “retrospective” (data collected after neonate had been discharged from the neonatal unit).[30]
- were conducted on neonatal sepsis. Neonates were defined as “under 28 days of life”.[31] Neonatal sepsis could include one or more of the following systemic infections: neonatal septicaemia/sepsis, pneumonia, meningitis, osteomyelitis, arthritis and urinary tract infections.[9] Definitions used in the included studies have been captured and reported in the results.
- reported laboratory-dependent (e.g. culture, immuno-haematologic, haematologic sepsis parameters) case definitions to confirm neonatal sepsis. Additionally, studies on neonatal pneumonia should have reported using radiological investigations for diagnosis.[9, 32] Data on clinical/ probable sepsis or studies which used clinical criteria exclusively to diagnose neonatal sepsis were excluded.
- reported on one or more risk factors of neonatal sepsis.

An outcome was included in the meta-analysis if at least two studies reported quantitative data for that outcome. These could be either crude numbers (events and non-events), odds ratio (OR; unadjusted or adjusted), or mean/median along with range/inter-quartile range/standard deviation (SD).

We excluded

- intervention studies, reviews, meta-analysis, commentaries and qualitative studies
- data/ studies using only clinical criteria for diagnosis of neonatal sepsis

### Study selection

Two authors (SM and MG) independently screened the studies on EndNote x7.8 in title, abstract and full-text stages. During full-text study selection, both the authors had to approve the study in order for it to be included in the review. Disagreements during full-text study selection were resolved by discussion and reaching consensus in the presence of senior authors (LL and VG).

### Data extraction and quality assessment

Two authors (SM and MG) extracted data and performed quality assessment of included studies using a pilot-tested form on Microsoft Excel 2016. Domains of extraction included characteristics of studies, methodological details, definitions, type of systemic infection, risk factors, confounding factors, funding and limitations. Crude data (dichotomous and continuous) for events and non-events, where available, was extracted and converted to OR.[33, 34] Otherwise, unadjusted and/or adjusted OR, Relative Risk (RR), p-value and CI were extracted and used. Authors of studies were contacted in an attempt to obtain missing information or gain clarity of information on methodology (e.g. study setting, case definition) and outcomes. If there was no authors’ reply or the reply was inadequate, that study/ outcome data was excluded from the review.

The Newcastle-Ottawa Scale (NOS) was used for quality assessment of case-control studies and cohort studies.[35] A modified version of NOS was used for cross-sectional studies. In addition, the National Heart, Lung, and Blood Institute’s (NHLBI) “Quality Assessment Tool for Observational Cohort and Cross-Sectional Studies”[36] was used. The outcome of this appraisal has been discussed in the narrative synopsis.

Disagreements during data extraction and quality assessment were resolved by discussion and consensus in the presence of senior authors (LL and VG).

### Data analysis and reporting

Data for meta-analysis was entered on MS Excel by one author (SM) and verified by another (MG). Meta-analysis was performed using RevMan v.5.3. Heterogeneity was assessed and reported using Chi^2^ test, I^2^ statistic and Tau^2^. An I^2^ value of 25–50% was considered as low, 50–75% as moderate and ≥75% as high heterogeneity.[37] The random-effects model was used for meta-analysis as substantial heterogeneity was anticipated in the methodology and definitions of sepsis and risk factors. [38, 39] The Dersimonian-Laird (DL) method was used primarily, the results of which have been presented and discussed in this paper. In addition, a random-effects meta-analysis using the Hartung-Knapp-Sidik-Jonkman (HKSJ) method was also performed as there were less than 10 studies in the meta-analysis. This method has been shown to be superior compared to the DL method for meta-analysis of intervention studies.[40] The results have been presented alongside those from DL method. Estimates were not pooled across the time of onset of sepsis (i.e. early vs late onset) or type of systemic infection (e.g. sepsis vs pneumonia vs meningitis). Effect sizes have been reported in OR for dichotomous data and standardized mean difference (SMD) for continuous outcomes. Wherever possible, transformation of raw data to OR, mean, SD and Standardized Mean Difference (SMD) was performed and used.[33, 34, 41] Pooled effect estimates were reported with 95% confidence intervals (CI). Forest plots were used to display the results graphically. We were able to perform subgroup analysis for only the following two of the four subgroups that were planned: a) case definition and (b) study design. The overall results of subgroup analysis have been provided in the results for the respective risk factor. A sensitivity analysis was performed by excluding one study at a time on OpenMeta-analyst.[42] The data used for these analyses are publicly available through Open Science Framework (URL: osf.io/465jx).

Tables and textual explanations have been provided wherever a meta-analysis was not possible. Additionally, the characteristics of studies, risk factor profile of included studies, and quality assessment results have been outlined in tables with a brief description. Funnel plots (using standard error and log OR) were used to assess any potential publication bias using Comprehensive Meta Analysis V3.3.070 (trial/ evaluation version).

## Results

**Fig 1:**
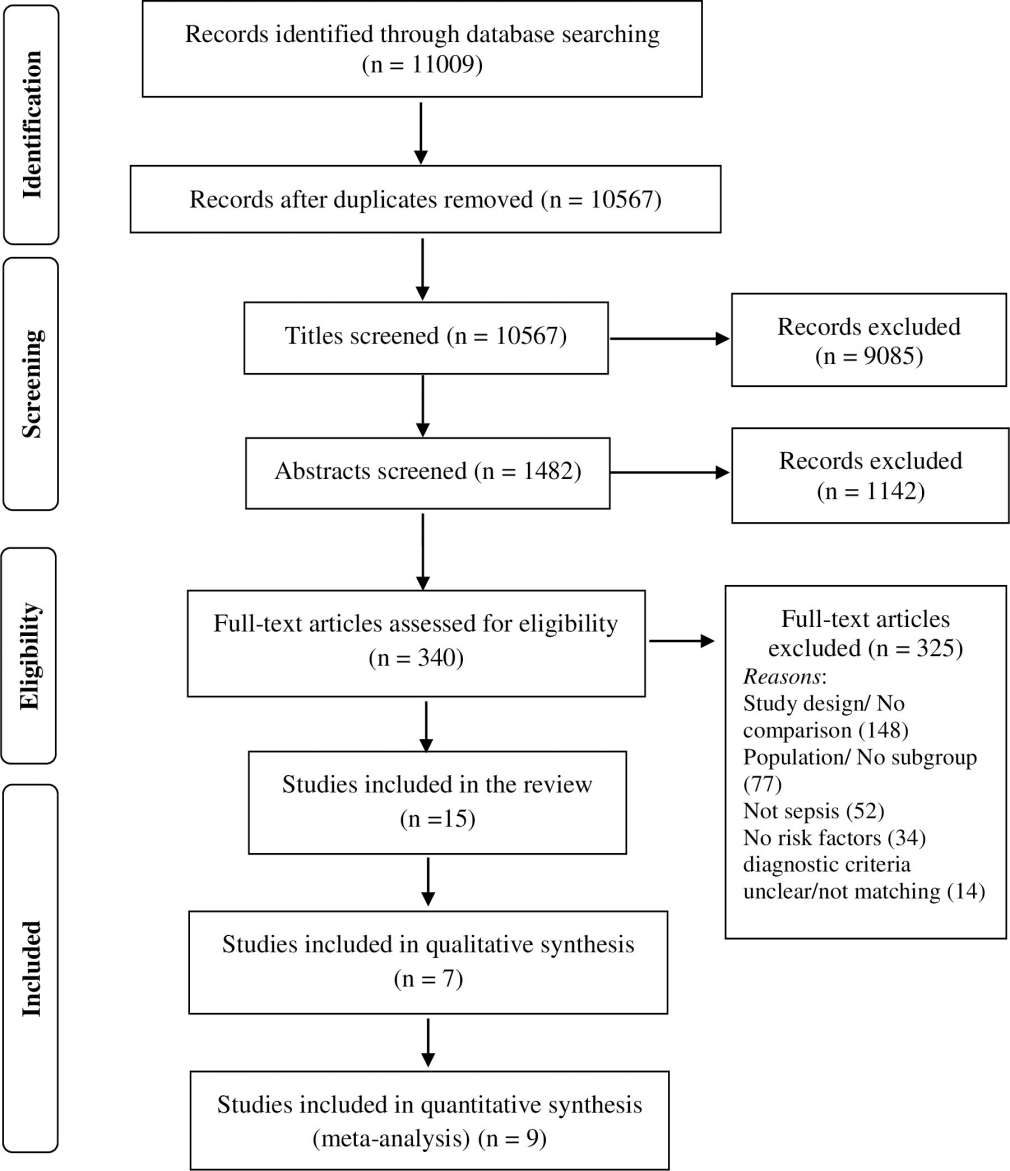
A PRISMA chart outlining the study selection results in our review.

A total after 10,567 titles were screened, after excluding 442 duplicate records. Of these, 9085 titles were excluded and 1482 abstracts were screened. Of these, 340 full text records were screened and 15 full texts met the inclusion criteria in our review, after discussion and consensus. Reasons for exclusion of 325 full-text records were lack of a comparison group/ wrong study design (n = 148), population not neonates/ no subgroup analysis (n = 77), infection not sepsis (n = 52), no risk factors studied (n = 34) and unclear/ wrong diagnostic criteria (n = 14). The study selection process is illustrated in Fig 1.

### Characteristics of included studies

Of the 15 included studies, nine studies were prospective (three cohort studies) and seven were retrospective (six case-control studies) in design. The total sample size was 53,224 (minimum:60;[43] maximum: 34,362[44]). The total sample sizes for case-control and cohort studies were 1076 (minimum: 60;[43] maximum: 546[45]) and 14,364 (minimum: 98;[46] maximum: 13,530[44, 47]) respectively. All studies were conducted in tertiary care hospitals and two were multi-centre studies.[47, 48] The minimum study duration was six months[43] and the maximum was 17 years.[45] Thirteen studies reported neonatal sepsis, two reported ventilator-associated pneumonia (VAP),[46, 49] and one reported neonatal meningitis.[44] Two studies reported exclusively on EOS,[45, 50] one of which focused on Group B Streptococcal (GBS) sepsis.[45] The characteristics of the included studies are provided in Table 1.

**Table 1:** Characteristics of included studies.

Sl. No	Study ID	Location	Study period	Setting	Neonate definition	Study design	Sample size	Risk Factor
Neonatal sepsis (diagnosis using haematologic sepsis parameters)								
1.	Bhakri2017[51]	Haryana	November 2014—October 2015	Neonatal division of rural tertiary care hospital	Not specified	Case-control	100	Maternal, Neonatal
2.	Das2016[52]	Odisha	March 2014—September 2015	NICU of private tertiary care teaching hospital	Not specified	Case-control	120	Neonatal
3.	Pradhan2016[53]	West Bengal	March 2012—March 2013	NICU of public tertiary care hospital	<28 days	Prospective observational	92	Neonatal
4.	Soni2013[54]	New Delhi	Not specified	NICU of public tertiary care hospital	Not specified	Prospective observational	90	Neonatal
5.	Verma2015[55]	Rajasthan	January—October 2014	NICU of public tertiary care hospital	Not specified	Prospective observational	3130	Neonatal
6.	DeNIS 2016a,b[44, 47]	New Delhi (multicentre)	July 2011 -February 2015	NICU of four public tertiary care hospitals	0–28 days of life	Prospective Cohort	13530	Maternal, Neonatal
Neonatal sepsis (culture-positive diagnosis)								
1.	Bhargava2017[56]	North India	Not specified	Tertiary care hospital	0–28 days	Case-control	100	Maternal, Neonatal
2.	Chaurasia2015[43],Fungal sepsis	Madhya Pradesh	January—June 2013	NICU of tertiary care hospital, autonomous	<28 days	Case-control	60	Maternal, Neonatal
3.	DeNIS 2016a,b[44, 47]	New Delhi (multicentre)	July 2011 -February 2015	NICU of four public tertiary care hospitals	0–28 days of life	Prospective Cohort	13530	Maternal, Neonatal
4.	Dutta2010[50] (early-onset sepsis)	North-West India	1 year	Level III neonatal unit, tertiary care teaching hospital	<72 hours of lifeª	Prospective Cohort	601	Maternal, Neonatal
5.	Prashant2013[57]	Not specified	Not specified	NICU of tertiary care hospital	Not specified	Case-control	150	Maternal, Neonatal
6.	Santhanam2017[45] (early-onset Group B Streptococcal sepsis)	Tamil Nadu	1998–2003, 1 January 2004–31 December 2014	Neonatology unit, tertiary care perinatal centre, private charitable/patient-paid	<72 hours of life^ª^	Case-control	546	Maternal, Neonatal
7.	Sundaram2009[58]	Northern India	1995–1998, 2001–2006	Neonatal unit of tertiary care hospital	Not specified	Prospective observational	34362	Neonatal
8.	Tapader2014[48]	New Delhi (1), West Bengal (2) (multicentre)	1-2007-2011, 2-2008-2009, 3-2009-2010	1. NICU & post-natal ward, tertiary care hospital 2. Microbiology, tertiary care hospital 3. SNCU, district hospital; all public sector	Not specified	Prospective observational	110	Maternal, Neonatal
Neonatal ventilator-associated pneumonia								
1.	Tripathi2010[46]	Not specified	September 2004—August 2005	NICU of tertiary care teaching hospital	CDC ^b^, NNIS^c^	Prospective cohort	98	Neonatal
2.	Vijayakanthi2015[49]	Tamil Nadu	1 January 2007–31 October 2007	NICU of public tertiary care hospital	CDC^b^	Retrospective cohort	135	Maternal, Neonatal
Neonatal meningitis								
1.	DeNIS 2016b[44]	New Delhi (multicentre)	July 2011 -February 2015	NICU of four public tertiary care hospitals	0–28 days of life	Prospective Cohort	13530	Maternal, Neonatal

Institute was classified as teaching and non-teaching only if mentioned in the article. Where setting was not specified, but institute name was provided, the details about the level of care and sector of hospital was found through an internet search of the institute’s name.

ªAuthors reported only EOS.

^b^CDC: Centers for Disease Control and Prevention.

^c^NNIS: National Nosocomial Infections Surveillance system.

The definitions reported in included studies have been summarized in S1 Table. There were variations in defining neonatal EOS and late-onset sepsis (LOS). Three studies defined EOS as less than 72 hours of life for neonatal sepsis[45, 50, 57] and as <5 days of mechanical ventilation for neonatal ventilator-associated pneumonia (VAP).[46] Similarly, there were variations in the case definitions and guidelines used to diagnose neonatal sepsis. Five studies reported the use of guidelines for defining/ diagnosing neonatal sepsis. Eight studies required culture-positive results to confirm neonatal sepsis.

### Risk factors

Based on the data available from included studies, factors were classified as neonatal and maternal factors in our review. Thirteen studies included both neonatal and maternal risk factors. The most frequently reported neonatal factor was birth weight (13 studies) and maternal factor was gestational age (12 studies). The factors reported in the included studies are summarized in S2 Table. A meta-analysis was performed for a total of eight factors. The details of factors included in the meta-analysis have been provided in Table 2.

**Table 2:** Risk factors included in meta-analysis for neonatal sepsis.

Outcome	Comparison	Studies	Sample size	Effect size	Pooled estimate	Heterogeneity I^2^, p-value
Sex	Male, Female	9	23753	Odds Ratio	1.31	49%, 0.05
Birthweight	<2500g, ≥2500g	5	44628	Odds Ratio	2.27	99%, <0.00001
	Mean, SD (grams)	3	282	Standardized Mean Difference	490.91	57%, 0.04
Resuscitation at birth	Yes, No	2	300	Odds Ratio	5.61	86%, 0.008
Need for artificial ventilation	Yes, No	3	270	Odds Ratio	18.39	0%, 0.73
Admission type	Inborn, Outborn	3	370	Odds Ratio	5.4	6%, 0.35
Gestational age	<37 weeks, ≥ 37 weeks	7	14557	Odds Ratio	2.05	77%, 0.0002
	Mean, SD (weeks)	2	182	Standardized Mean Difference	2.12	82%, 0.02
Mode of delivery	Vaginal Delivery, Caesarean delivery	5	570	Odds Ratio	2.13	87%, <0.00001
Premature Rupture of Membranes	Yes, No	3	349	Odds Ratio	11.14	0%, 0.54

In addition to the DerSimonian-Laird method, the Hartung-Knapp-Sidik-Jonkman method of random meta-analysis was used to pool the results, and are provided in Table 3. Six factors (male gender, outborn admission, need for artificial ventilation, birth weight, delivery <37 weeks of gestation, premature rupture of membranes) were significant when the DL method of random-effects meta-analysis was conducted. On using the HKSJ method, only three of these factors (need for artificial ventilation, delivery <37 weeks of gestation, premature rupture of membranes) retained significance. The results from the DL method have been reported in detail and discussed below in the paper.

**Table 3:** Results of random effects meta-analysis using DerSimonian-Laird method and Hartung-Knapp-Sidik-Jonkman methods.

Factor	DerSimonian-Laird (DL) method				Hartung-Knapp-Sidik-Jonkman (HKSJ) method			
	Pooled effect estimate	95% LCI	95% UCI	p value	Pooled effect estimate	95% LCI	95% UCI	p value
**Neonate-related**								
Male gender	1,31	1,02	1,68	0,03^a^	1,31	0,93	1,84	0,10
Outborn admission	5,46	2,39	12,49	<0,00001^a^	5,46	0,89	33,49	0,06
Low birth weight	2,27	0,51	10,09	0,28	2,27	0,4	12,94	0,26
Resuscitation at birth	5,61	0,61	51,79	0,13	5,61	0	1016	0,37
Need for artificial ventilation	18,39	8,21	41,18	<0,0000^a^	18,39	6,86	49,28	0,006^a^
Birth weight	-0,8	-1,25	-0,35	0,0005^a^	-0,8	-1,8	0,2	0,075
**Mother-related**								
Delivery <37 weeks of gestation	2,05	1,4	2,99	0,0002^a^	2,05	1,15	3,66	0,023 ^a^
Vaginal delivery	2,13	0,68	6,62	0,19	2,13	0,41	11,08	0,27
Premature rupture of membranes	11,14	5,54	22,38	<0,00001^a^	11,14	3,35	37	0,01^a^
Gestational age at delivery	-0,6	-1,42	0,23	0,16	-0,6	-5,94	4,74	0,39

^a^ significant (p value <0.05)

#### Data included in meta-analysis

**Fig 2:**
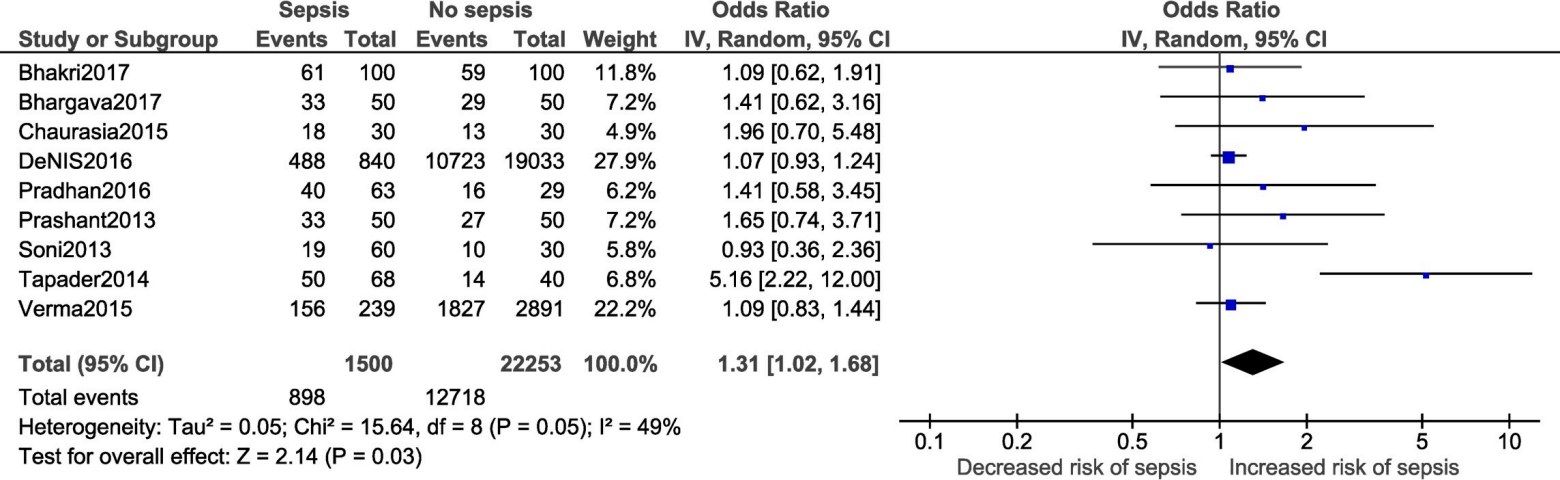
Forest plot showing a random-effects meta-analysis of male neonates with and without sepsis.

**Fig 3:**
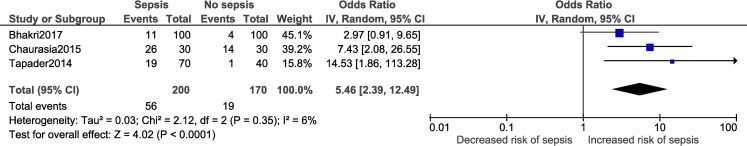
Forest plot showing a random-effects meta-analysis of outborn neonates with and without sepsis.

**Fig 4:**
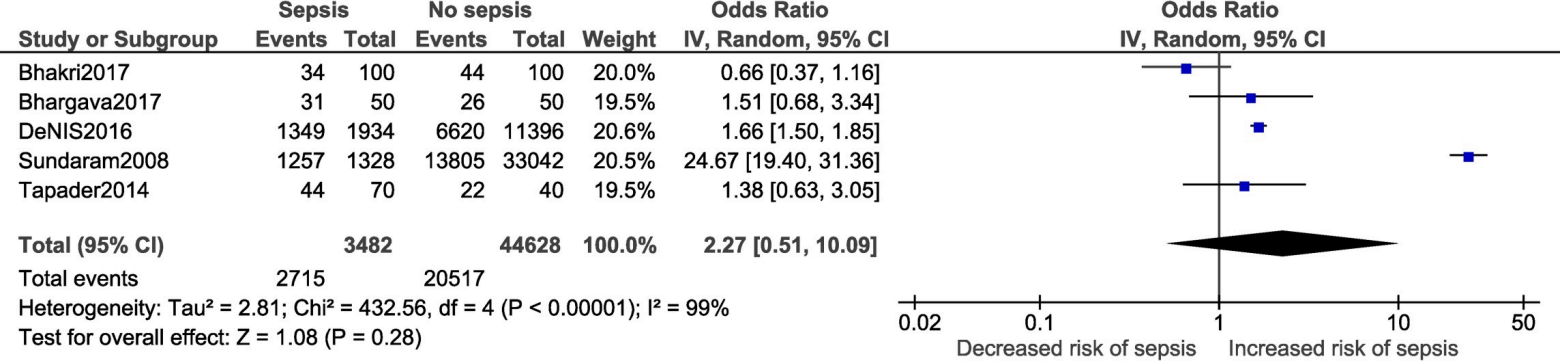
Forest plot showing a random-effects meta-analysis of low birth weight neonates with and without sepsis.

**Fig 5:**
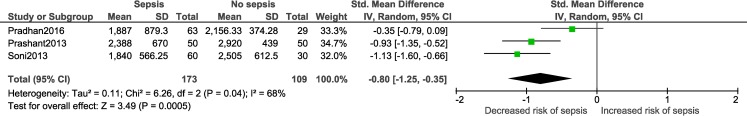
Forest plot showing a random-effects meta-analysis of standardized mean difference of birth weight of neonates with and without sepsis.

**Fig 6:**
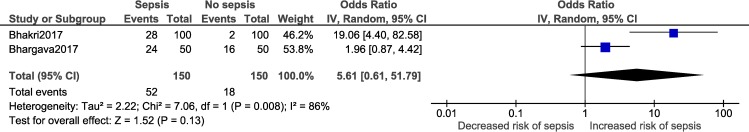
Forest plot showing a random-effects meta-analysis of neonates resuscitated at birth, with and without sepsis.

**Fig 7:**
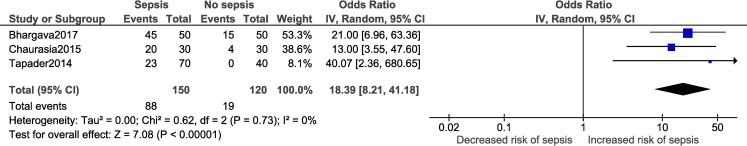
Forest plot showing a random-effects meta-analysis of neonates requiring artificial ventilation, with and without sepsis.

Neonatal factors:

A meta-analysis was performed for five risk factors (Figs 2–7), of which male sex (9 studies- OR: 1.3, 95% CI: 1.02, 1.68; I^2^ = 49%), outborn neonates (3 studies- OR: 5.5, 95% CI: 2.39, 12.49; I^2^ = 6%) and the need for artificial ventilation (3 studies- OR: 5.61; 95% CI: 8.21, 41.18; I^2^ = 0) significantly increased the odds of neonatal sepsis. Factors which increased the likelihood of sepsis among neonates, but were not significant in the meta-analysis, included low birth weight (5 studies- OR: 2.27; 95% CI: 0.51, 10.09; I^2^ = 99%) and resuscitation at birth (2 studies- OR: 5.61; 95% CI: 0.61, 51.79; I^2^ = 86%).

**Fig 8:**
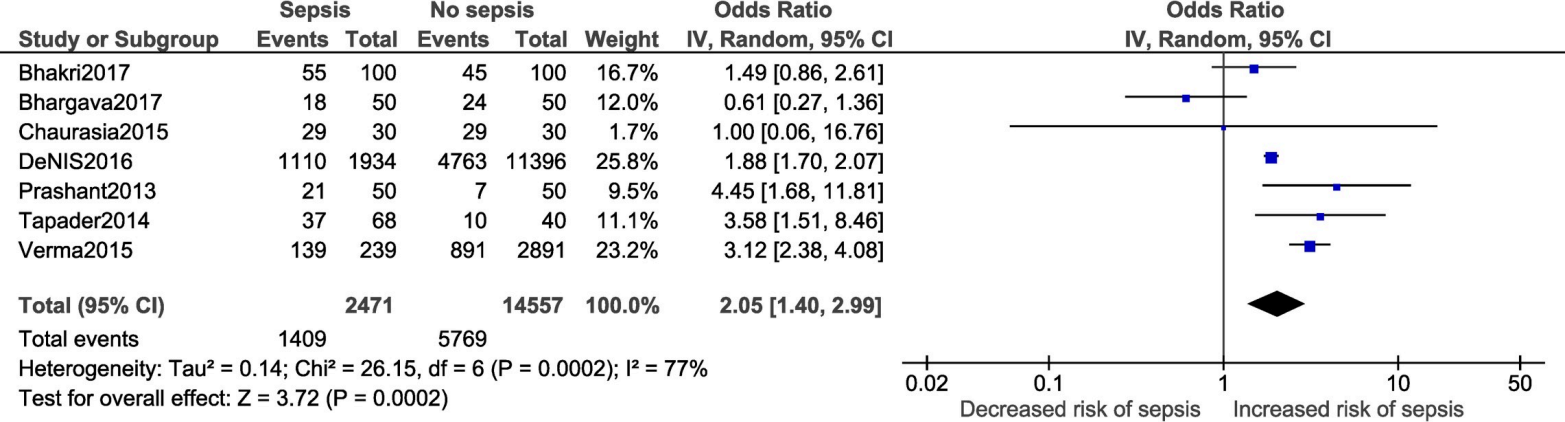
Forest plot showing a random-effects meta-analysis of neonates, with and without sepsis, born to mothers who delivered at <37 weeks of gestation.

**Fig 9:**
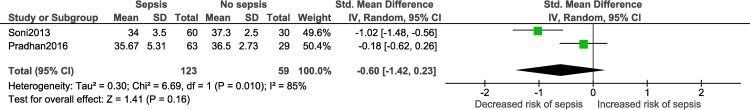
Forest plot showing a random-effects meta-analysis of gestational age of mothers of neonates with and without sepsis.

**Fig 10:**
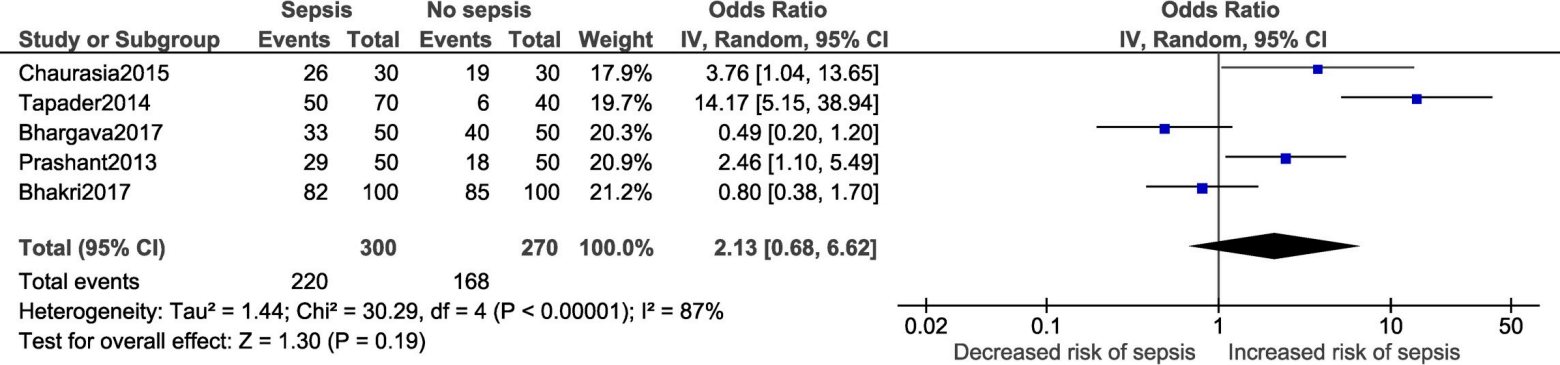
Forest plot showing a random-effects meta-analysis of neonates, with and without sepsis, born to mothers who had vaginal delivery.

**Fig 11:**
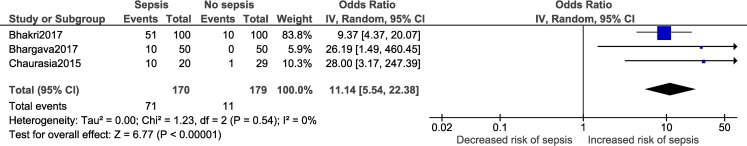
Forest plot showing a random-effects meta-analysis of neonates, with and without sepsis, born to mothers with premature rupture of membranes.

Maternal factors:

A meta-analysis was performed for three factors (Figs 8–11): gestational age (dichotomous; 7 studies), gestational age (continuous; 2 studies), mode of delivery (5 studies) and premature rupture of membranes (PROM; 3 studies). Gestational age<37 weeks (OR: 2.05; 95% CI:1.40, 2.99; I^2^ = 77%) and PROM (OR:11.14, 95% CI: 5.54, 22.38; I^2^ = 0) were associated with a significantly higher odds of neonatal sepsis.

**Fig 12:**
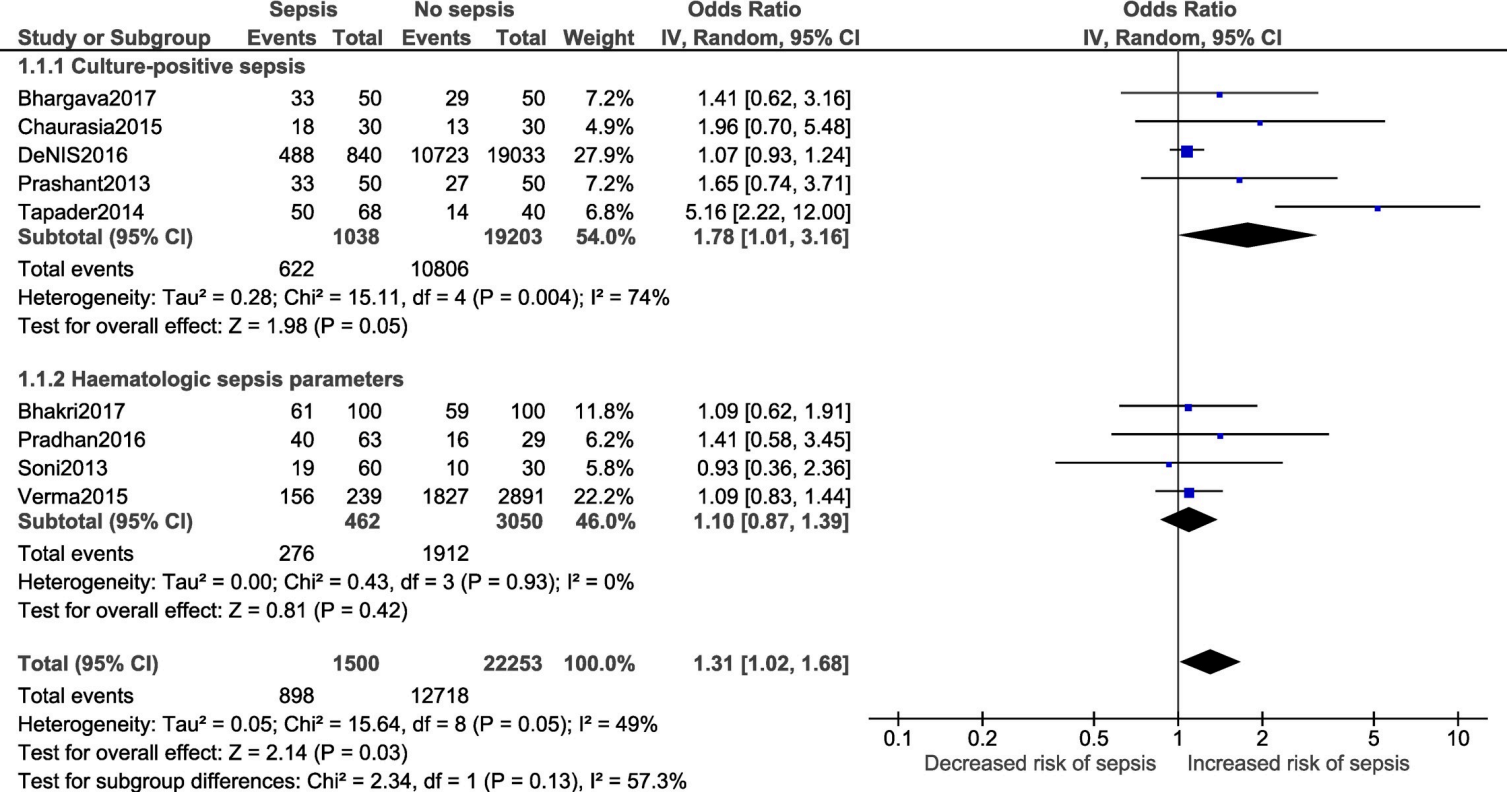
Forest plot showing random-effects meta-analysis for male neonates with and without sepsis sub-grouped by sepsis diagnostic criteria (9 studies) [IV: Inverse Variance; CI: Confidence Interval].

**Fig 13:**
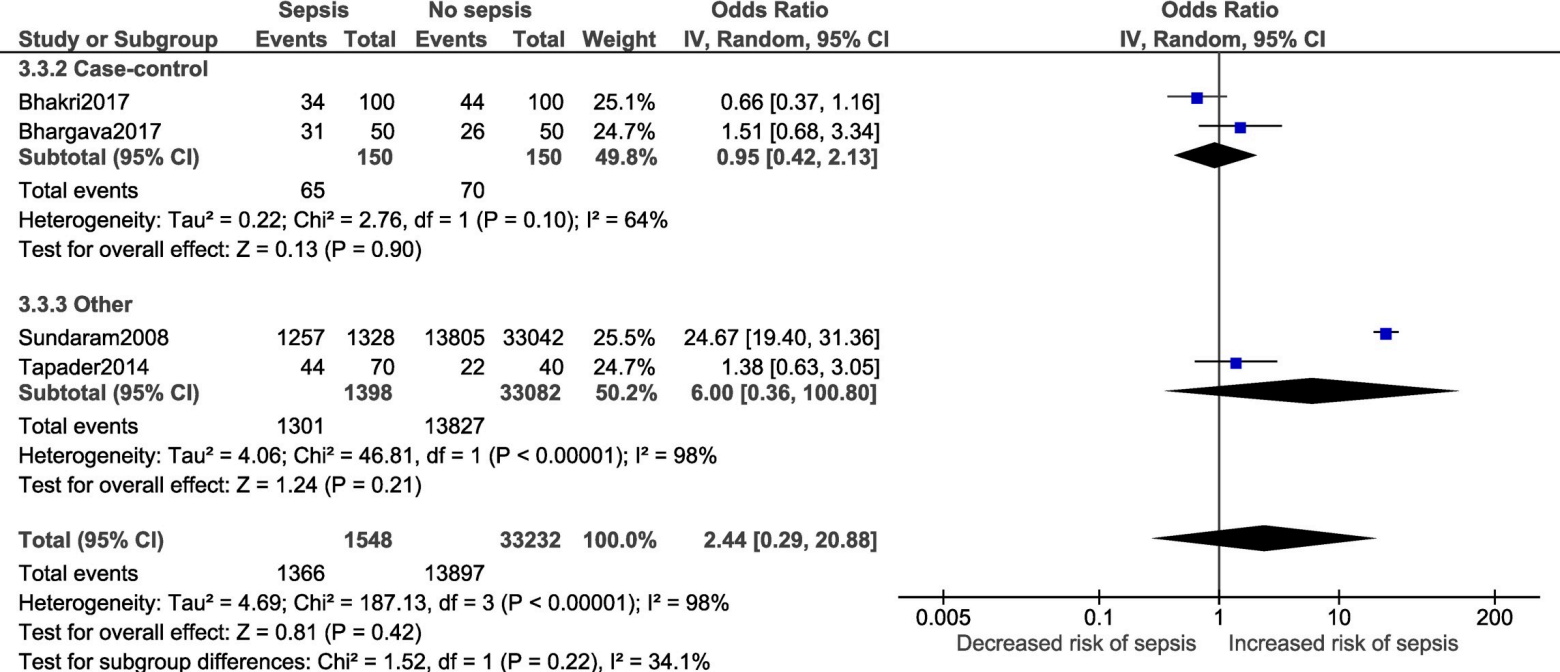
Forest plot showing random-effects meta-analysis for low birth weight (< 2500 grams) neonates with and without sepsis, sub-grouped by study design (4 studies) [IV: Inverse Variance; CI: Confidence Interval].

**Fig 14:**
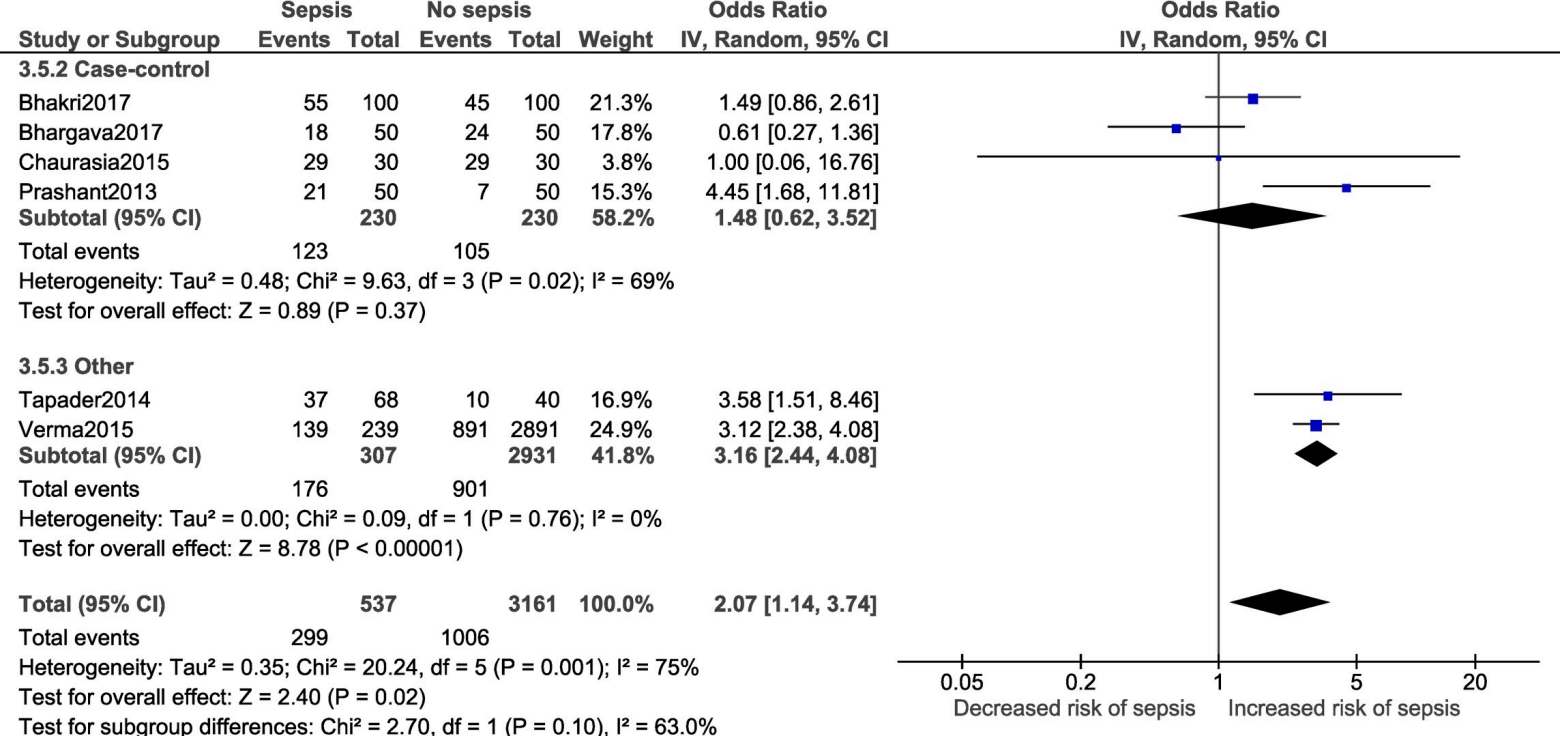
Forest plot showing random-effects meta-analysis of neonates, with and without sepsis, born to mothers delivering <37 weeks of gestation, sub-grouped by study design (6 studies) [IV: Inverse Variance; CI: Confidence Interval].

**Fig 15:**
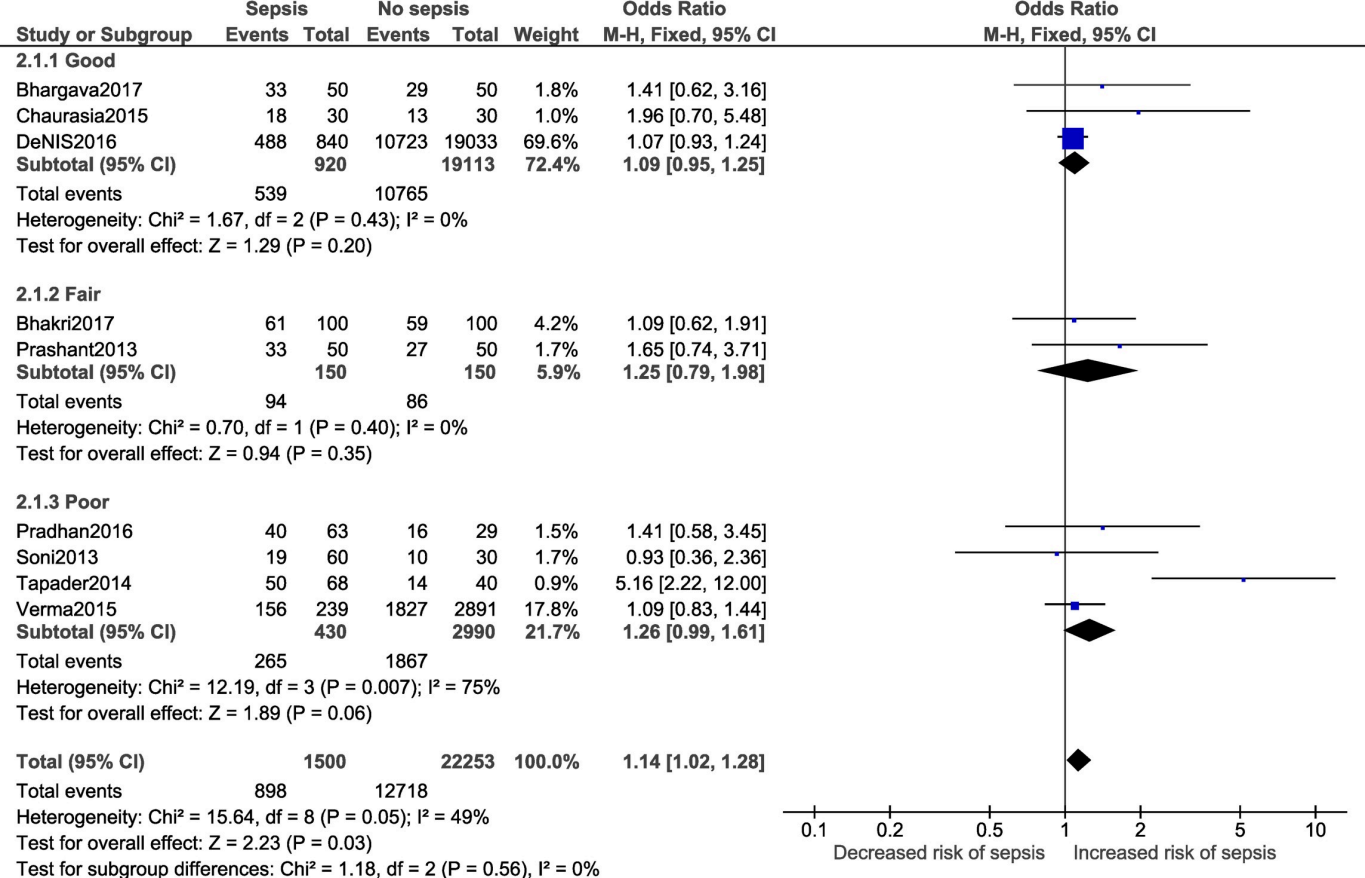
Forest plot showing random-effects meta-analysis of male neonates, with and without sepsis, sub-grouped by study quality (9 studies) [IV: Inverse Variance; CI: Confidence Interval].

**Fig 16:**
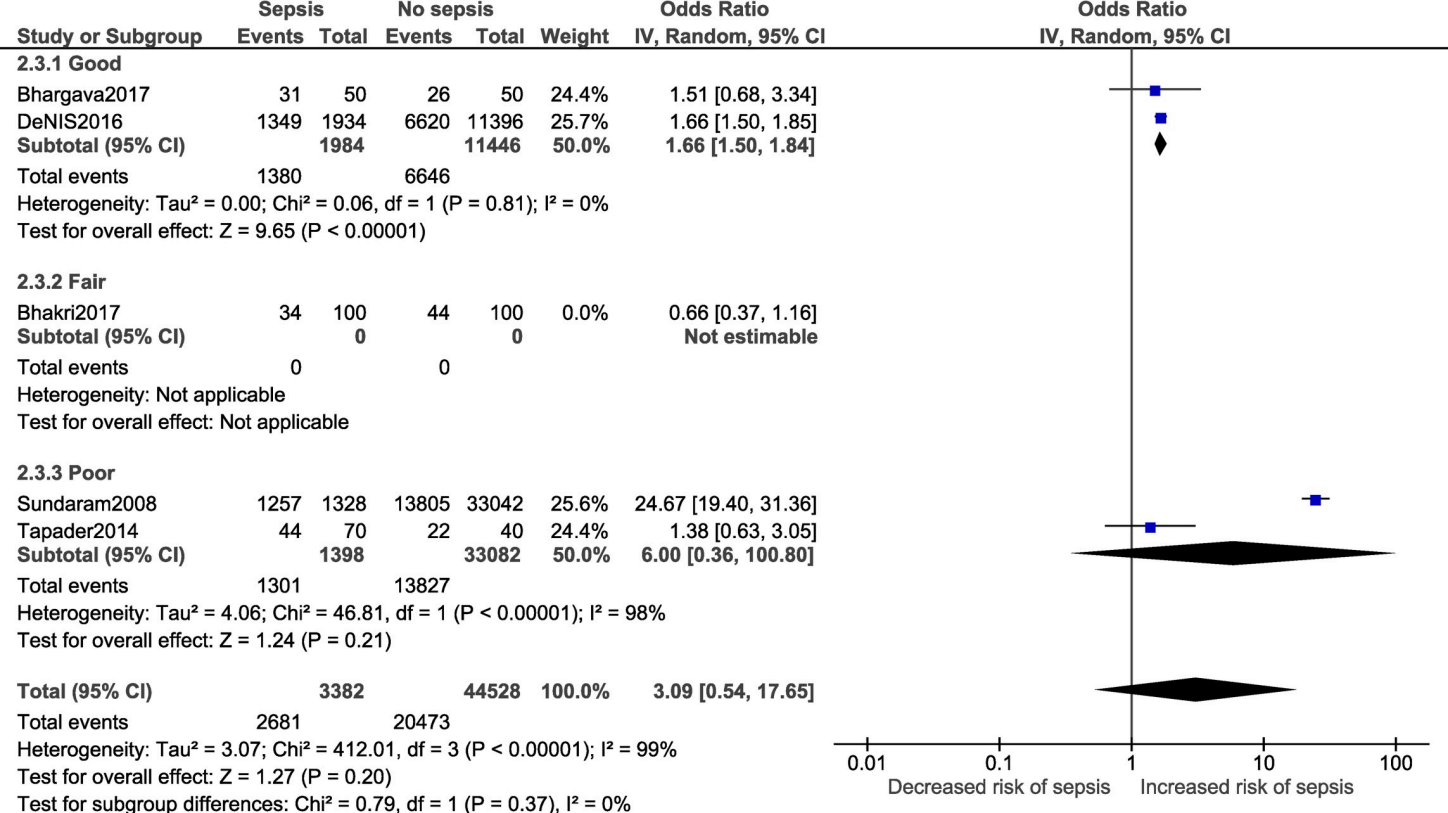
Forest plot showing random-effects meta-analysis for low birth weight (< 2500 grams) neonates with and without sepsis, sub-grouped by study quality (4 studies) [IV: Inverse Variance; CI: Confidence Interval].

**Fig 17:**
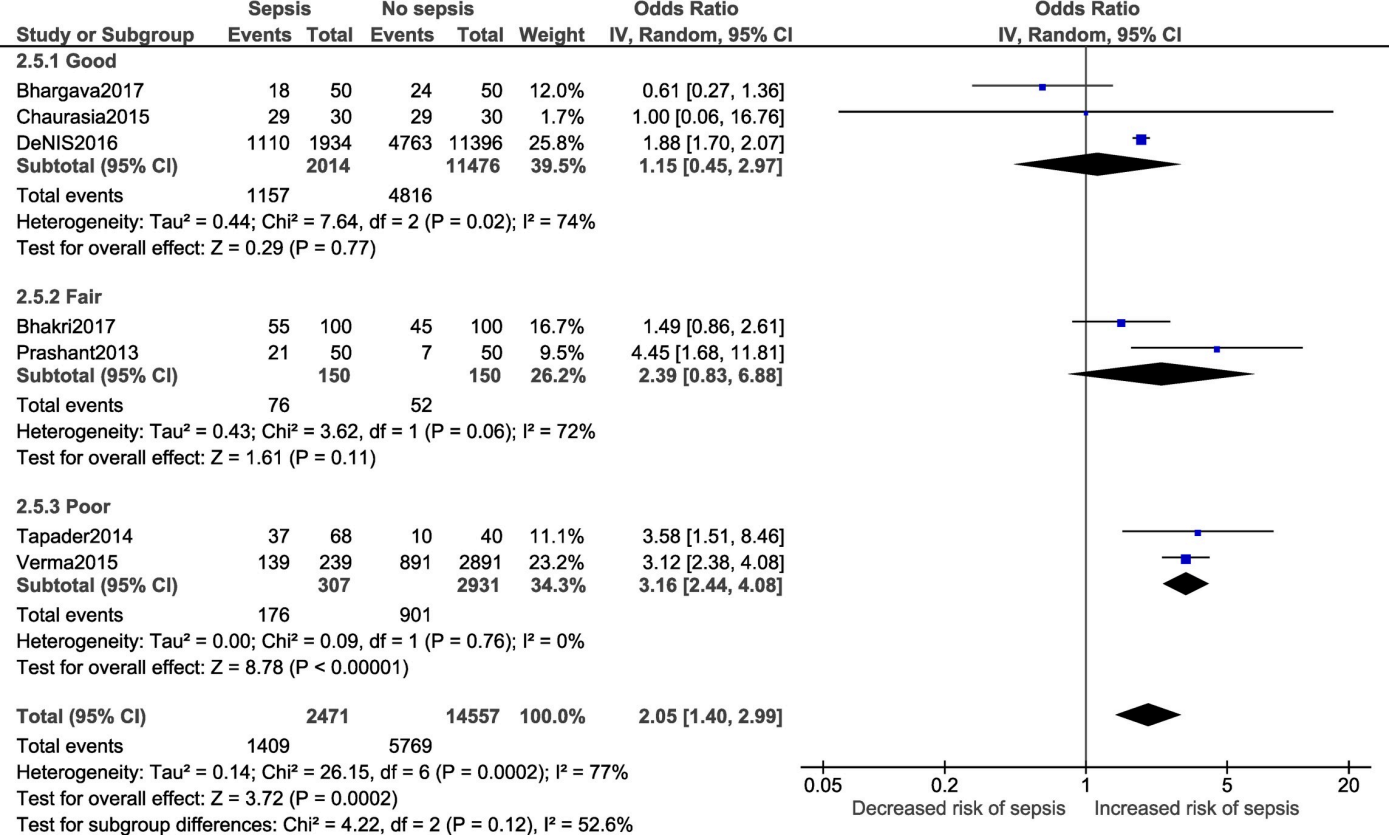
Forest plot showing random-effects meta-analysis of neonates, with and without sepsis, born to mothers delivering <37 weeks of gestation, sub-grouped by study quality (7 studies) [IV: Inverse Variance; CI: Confidence Interval].

**Fig 18:**
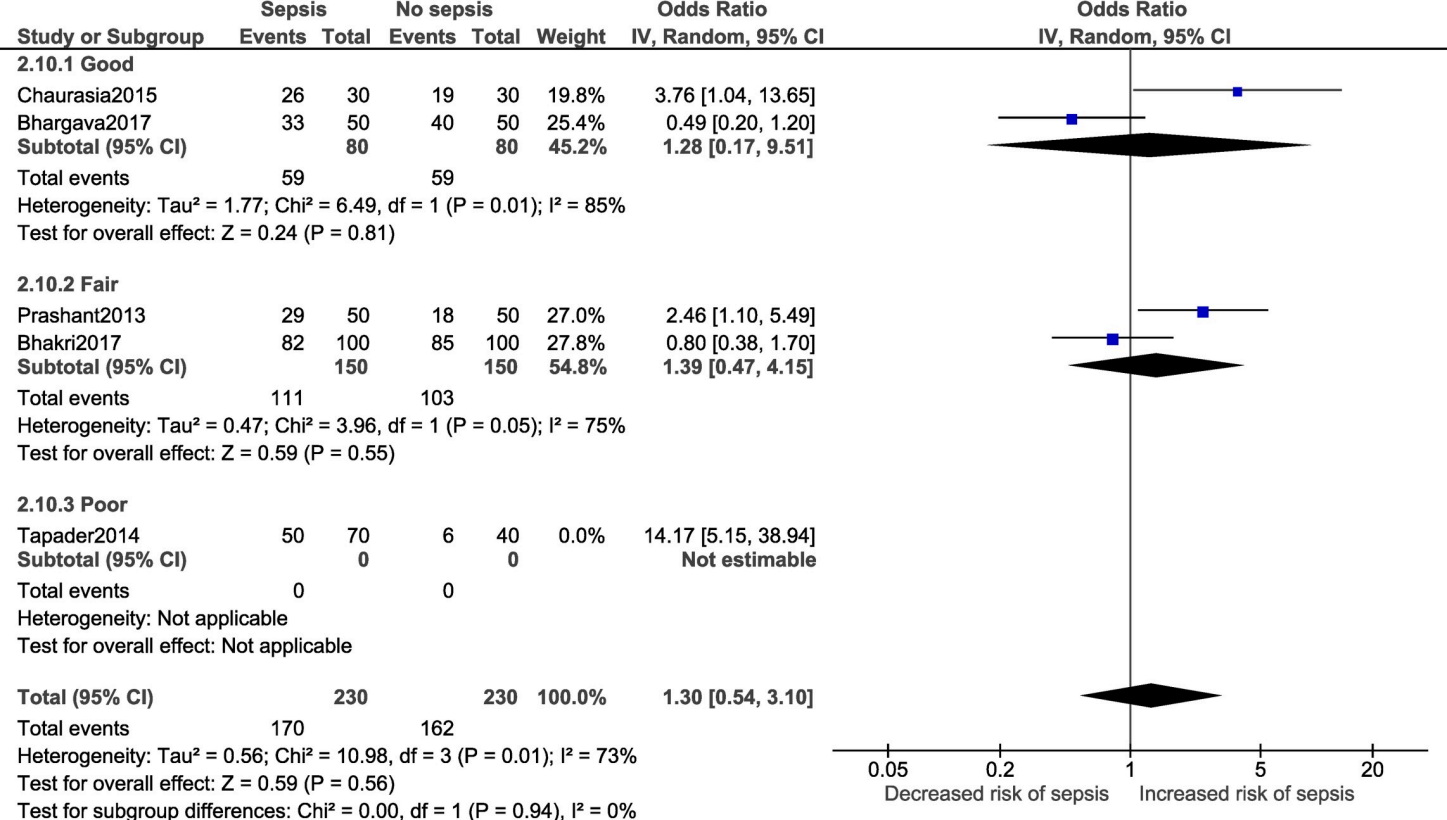
Forest plot showing random-effects meta-analysis of neonates, with and without sepsis, born to mothers who delivered vaginally, sub-grouped by study quality (4 studies) [IV: Inverse Variance; CI: Confidence Interval].

Subgroup analysis:

Adequate number of studies (two or more) to perform a subgroup analysis was available for a total of four outcomes i.e. male gender, low birth weight, delivery <37 weeks of gestation and vaginal delivery. Forest plots for the subgroup analysis have been provided in S1 Forest Plots. Subgroup analysis based on diagnostic criteria did not reveal any significant differences in the pooled estimate for the neonatal factors (Fig 12). Subgroup analysis based on study design for low birth weight (Fig 13; p = 0.22, I^2^ = 34.1%) and delivery <37 weeks of gestation (Fig 14; p = 0.10, I^2^ = 63%) did not reveal overall significant differences. For both factors (i.e. low birth weight and delivery <37 weeks of gestation), case-control studies showed a lower and non-significant pooled estimate value (low birth weight: OR: 0.95, 95% CI: 0.42, 2.13; delivery <37 weeks of gestation: OR: 1.48, 95% CI: 0.62, 3.52) compared to other observational study designs. Subgroup analysis (Figs 15–18) by study quality did not reveal significant differences overall for any of the four outcomes. Studies rated as ‘good’ quality consistently showed lower pooled ORs compared to those rated as ‘fair’ or ‘poor’, for all outcomes.

Additionally, the results of the subgroup analysis performed using both the DL and the HKSJ methods have been presented in Table 4.

**Table 4:** Results of subgroup analysis using DerSimonian-Laird (DL) and Hartung-Knapp-Sidik-Jonkman (HKSJ) methods of random-effects meta-analysis.

Factor and subgroup	Method used	Pooled OR	95% CI (lower)	95% CI (upper)	p value
**A. By diagnostic criteria**					
*Male gender*					
Culture-positive	DL method	1.78	1.01	3.16	0.05
	HKSJ method	1.78	0.85	3.74	0.09
Haematologic sepsis paramaters	DL method	1.10	0.87	1.39	0.42
	HKSJ method	1.10	0.9	1.34	0.23
Overall	DL method	1.31	1.02	1.68	0.03
	HKSJ method	1.31	0.94	1.83	0.10
**B. By study design**					
*Low birth weight*					
Case-control study	DL method	0.95	0.42	2.13	0.90
	HKSJ method	0.95	0	188.54	0.92
Other study designs	DL method	6	0.36	100.80	0.21
	HKSJ method	6	0	5.41E+08	0.43
Overall	DL method	2.44	0.29	20.88	0.42
	HKSJ method	2.44	0.19	31.35	0.35
*Delivery <37 weeks of gestation*					
Case-control	DL method	1.48	0.62	3.52	0.37
	HKSJ method	1.48	0.37	5.92	0.43
Other study designs	DL method	3.16	2.44	4.08	<0.00001
	HKSJ method	3.16	1.14	8.73	0.044
Overall	DL method	2.07	1.14	3.74	0.02
	HKSJ method	2.07	0.92	4.63	0.068
**C. By study quality**					
*Male gender*					
Good	DL method	1.09	0.95	1.25	0.20
	HKSJ method	1.09	0.85	1.4	0.28
Fair	DL method	1.25	0.79	1.98	0.35
	HKSJ method	1.25	0.11	13.73	0.45
Poor	DL method	1.26	0.99	1.61	0.06
	HKSJ method	1.26	0.69	2.3	0.31
Overall	DL method	1.14	1.02	1.28	0.03
	HKSJ method	1.14	0.99	1.32	0.07
*Low birth weight*					
Good	DL method	1.66	1.50	1.84	< .00001
	HKSJ method	1.66	0.72	3.84	0.08
Poor	DL method	6	0.36	100.80	0.21
	HKSJ method	6	0	5.4E+08	0.43
Overall	DL method	3.09	0.54	17.65	0.20
	HKSJ method	3.09	0.33	29.02	0.21
*Delivery <37 weeks of gestation*					
Good	DL method	1.15	0.45	2.97	0.77
	HKSJ method	1.15	0.23	5.77	0.74
Fair	DL method	2.39	0.83	6.88	0.11
	HKSJ method	2.39	0	2046.32	0.35
Poor	DL method	3.16	2.44	4.08	< .00001
	HKSJ method	3.16	0	488084	0.44
Overall	DL method	2.05	1.40	2.99	0.0002
	HKSJ method	2.05	1.15	3.66	0.02
*Vaginal delivery*					
Good	DL method	1.28	0.17	9.51	0.81
	HKSJ method	1.28	0	499778	0.85
Fair	DL method	1.39	0.47	4.15	0.55
	HKSJ method	1.39	0	1745.9	0.66
Overall	DL method	1.30	0.54	3.10	0.56
	HKSJ method	1.30	0.3	5.66	0.61

Sensitivity analysis:

Overall, leave-one-out meta-analysis revealed that male gender and birth weight did not retain significance when single studies were excluded. An increase in the pooled effect estimates and uncertainty was observed for outborn admission, the need for artificial ventilation and premature rupture of membranes on excluding single studies during sensitivity analysis. The detailed results, including the forest plots, of the sensitivity analyses have been reported in S2 File.

**Fig 19:**
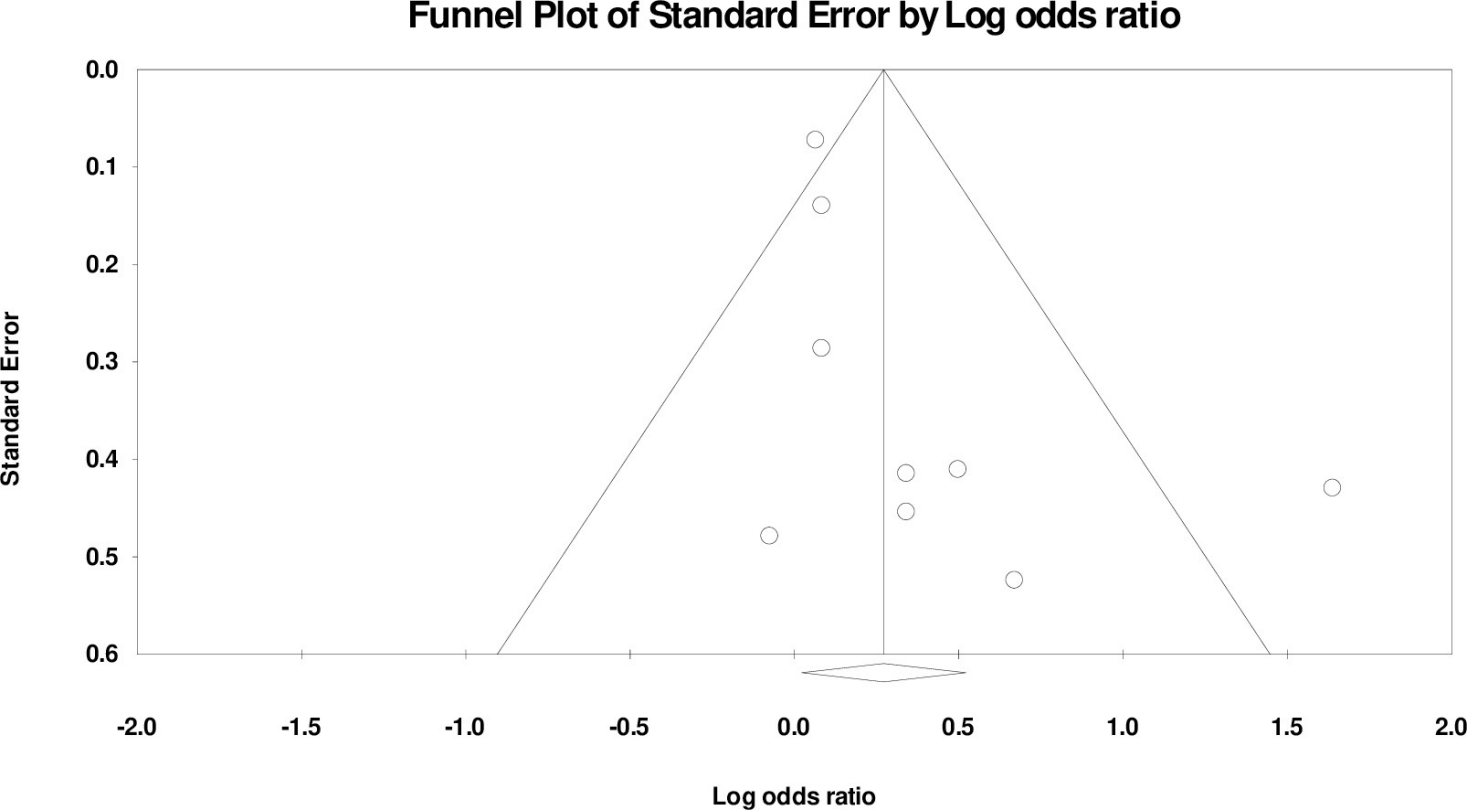
Funnel plot illustrating publication bias assessment of male gender as a risk factor of neonatal sepsis.

**Fig 20:**
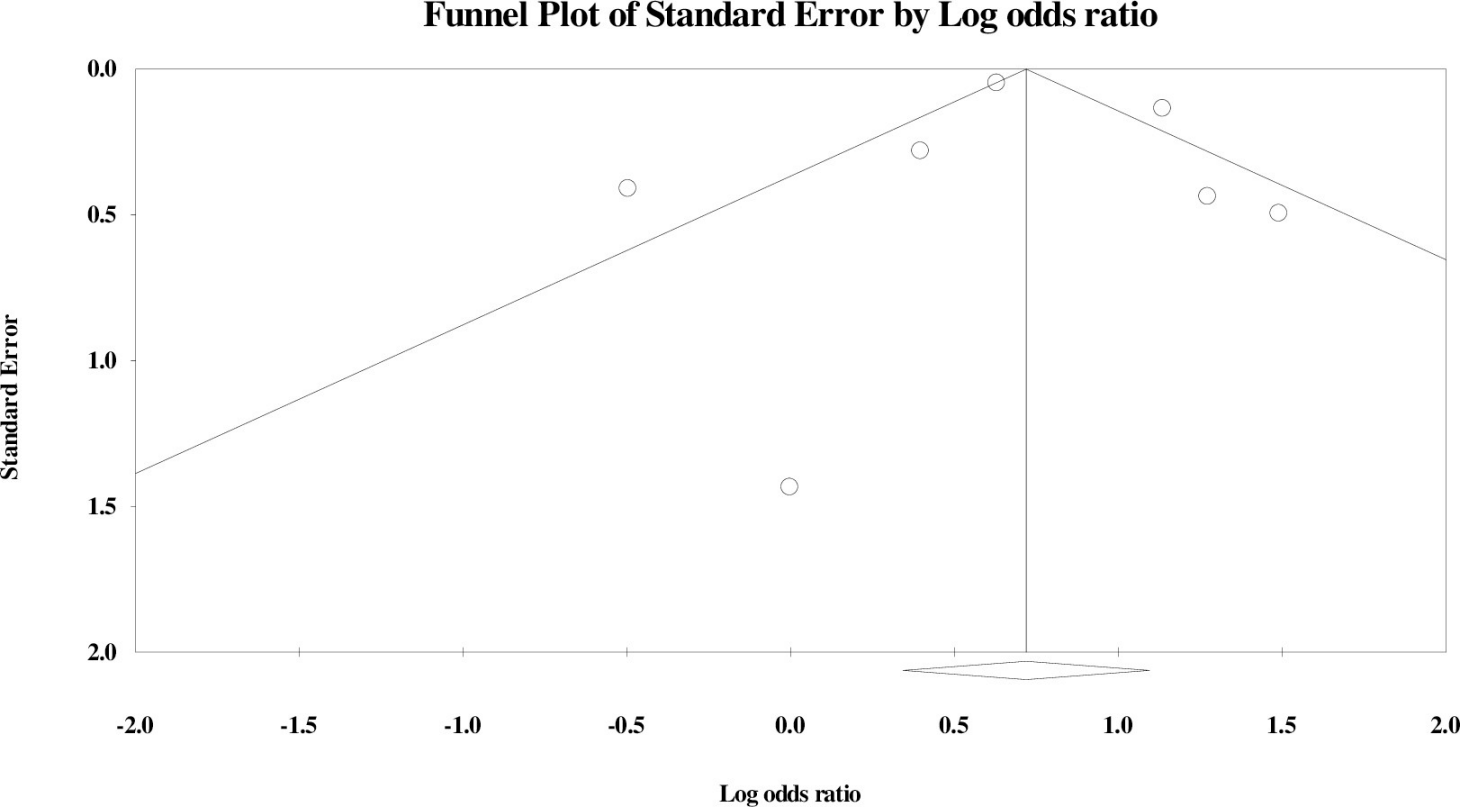
Funnel plot illustrating publication bias assessment for delivery <37 weeks of gestation as risk factor for neonatal sepsis.

Publication bias:

Publication bias was assessed for male gender and delivery <37 weeks of gestation (see S1 Funnel Plots). On visual inspection, asymmetry was observed in the funnel plots of both the outcomes. Results from Egger’s regression test, however, did not show statistical significance for association between male gender (Fig 19; 9 studies, p = 0.08) or delivery <37 weeks of gestation (Fig 20; 7 studies, p = 0.83) with neonatal sepsis.

#### Data not included in meta-analysis

Factors for which a meta-analysis was not possible, and were significantly associated with neonatal sepsis, meningitis and VAP (summarized in S3 Table) included:

- Neonatal sepsis: low vitamin D level (serum 25(OH)D),[52] expressed/formula feed,[56] congenital anomalies,[56] use of intravenous fluids,[47] insertion of venous catheter,[56] and length of hospitalization and prior antibiotic use >7 days (fungal sepsis),[43] multiple PV examination,[51] and antenatal history (e.g. prior antibiotic use, previous surgery).[51, 56]
- Neonatal VAP: very low birth weight,[46] prolonged mechanical ventilation,[46] prolonged hospitalization[46] and multiple reintubations (>1).[49]
- Neonatal meningitis: birth weight <2.5 kg, gestational age <37 weeks.[44]
- Factors included in single studies and were found to be not significant included:
- Neonatal sepsis: birthweight <1.5 kg (fungal sepsis),[43] small-for-gestational age,[51] delayed enteral feed and insertion of venous catheter (gram-positive sepsis),[56] congenital anomalies (gram-negative sepsis),[56] prolonged labour (fungal sepsis),[43] primigravida,[51] maternal infection[56] or febrile illness (fungal sepsis),[43] foul-smelling liquor (fungal sepsis),[43] antenatal steroid,[56] and place of living (fungal sepsis).[43]
- Neonatal VAP: Postnatal age,[46] sex of the neonate,[46] prolonged hospitalization/ level III stay,[49] small-for-gestational age,[46] respiratory distress[49] or unstable cardiopulmonary status at admission,[49] resuscitation at birth,[46] repeated reintubations,[46] preterm delivery,[46] premature rupture of membranes,[46] and home delivery,[49] for neonatal VAP.

### Quality assessment

On performing quality assessment (see S4 Table) using the NOS, five studies each were rated as good (two case-control [43, 56] and three cohort studies [46, 47, 50]), fair (four case-control [45, 51, 52, 57] and one cohort study [49]) and poor (all cross-sectional studies [48, 53, 54, 55, 58]). The domain with the lowest rating was ‘comparability’ for case-control studies, ‘outcome’ assessment for cohort studies and ‘confounder’ assessment for cross-sectional studies. Similarly, on the NHLBI scale, the low scoring components of the studies included sample size justification, exposure measurement and assessment, assessment and adjustment of confounding variables, and blinding of outcome assessors. Overall, of the nine studies assessed by NHLBI scale, four studies were rated as fair,[53–55, 58] three studies were rated as good [44, 46, 50] and two studies were rated as poor quality.[48, 49] None of the studies reported using a reporting checklist to report their studies.

## Discussion

In a “time-critical clinical course” like that of sepsis,^1^ knowledge of risk factors aids in early prediction, identification and timely empirical antibiotic therapy, which are key to reducing neonatal morbidity and mortality.[7, 15] Male gender, outborn admissions, need for artificial ventilation, gestational age <37 weeks and PROM were found to significantly increase the odds of neonatal sepsis in our meta-analysis. Due to limited number of studies, we were unable to find conclusive evidence for the timing of onset of the systemic infection, and for systemic infections other than sepsis in neonates (e.g. pneumonia, meningitis).

### Definitions

There was variation in the case definitions and guidelines used to diagnose neonatal sepsis in studies included in our review, considering a lack of global consensus on the same. Similar variability has been found in global literature on neonatal sepsis.[11, 59] Applicability of definitions and management guidelines for neonatal sepsis adopted by HICs have limitations in LMICs due to epidemiological differences[60] and inadequate infrastructure (e.g. laboratory setups) in resource-limited settings.[5, 13] Variations in definitions have direct implications on research, and clinical practice, and reflect on achieving the overall aim of reducing burden of sepsis in neonates.[10, 11, 61] While reaching a consensus is not in any way an easy task,[11] we encourage authors of primary studies to report guidelines which have influenced their case definitions for transparency and clarity, thus improving comparability. Use of reporting checklists (e.g. Strengthening the Reporting of Observational Epidemiology (STROBE)[61] and its recent extension for “Newborn Infection” (STROBE-NI)),[62] help ensure the complete and accurate reporting of studies.

### Risk factors

A higher incidence of sepsis has been suggested among male neonates, possibly based on the “male disadvantage hypothesis”.[63–66] Males neonates are more sensitive to adverse perinatal and postnatal environmental conditions, and are more likely to be born preterm and with a lower birth weight, both of which increase the risk of neonatal sepsis.[65] Additionally, more initial respiratory support required by male neonates may lead to poorer outcomes.[67]

Maternal factors such as premature delivery (gestational age <37 weeks) and PROM have also been implicated as significant risk factors in a meta-analysis on neonatal EOS (OR: 2.3, 95% CI: 1, 5.4; I^2^ = 93.4%; aOR: 4.9, 95% CI: 1.9, 12.8),[2] and a critical literature review with secondary analysis on neonatal early-onset GBS sepsis (preterm delivery OR: 4.83; PROM OR: 9.74).[23] The latter also found low birth weight (LBW; OR: 7.37, 95% CI: 4.48, 12.1) to be a significant risk factor of neonatal early-onset GBS sepsis. Though LBW increased the odds of neonatal sepsis in our review (OR: 2.27), this was not significant. A possible explanation for this might be that the former only included data from the USA; we found a limited number of studies of LBW with substantial heterogeneity (I^2^ = 99%) in our meta-analysis. Additionally, the variations in effect size found in the discussed literature may be explained by the reviews’ broader inclusion criteria (e.g. inclusion of global studies and randomized controlled trials, case definition variations) and their focus on EOS infections.

Additionally, reviews have found maternal colonization/infection,[2, 23, 24] prolonged rupture of membranes >18 hours,[2, 23] and intrapartum antibiotic prophylaxis[24] to significantly increase the risk of early-onset neonatal infections. Neonates are at a high risk of EOS, which can occur as a result of a direct transmission of the maternal colonizers (e.g. bacteria in the maternal vaginal tract) to the newborns during delivery.[2, 68] Intrapartum antibiotic prophylaxis has been recommended as an effective practice for at-risk mothers to reduce EOS globally.[17, 23, 26] In India, it is recommended for culture-positive mothers due to a very low prevalence of maternal GBS infection.[68]

Similarly, due to the limited number of studies and data from these studies, we did not conduct a meta-analysis for factors associated with neonatal VAP. Individually, studies found that prolonged mechanical ventilation, very low birth weight, repeated intubations and unstable cardiopulmonary status at admission independently increase the odds of VAP in neonates. A meta-analysis found, in addition to these factors, that the length of neonatal intensive care unit (NICU) stay, bronchopulmonary dysplasia, enteral feeding and parental nutrition increased the risk of neonatal VAP.[21]

Many factors in our meta-analysis had substantial heterogeneity, which may be explained by the population (e.g. type of controls, twins, inborn neonates), criteria used to define and/or diagnose sepsis (e.g. culture-dependant vs hematologic sepsis paramaters), definitions of risk factors and hospital policies in place (e.g. intrapartum antibiotic prophylaxis). Clarity on study design for 40% of the included studies was limited by incomplete reporting. Though subgroup analyses did not show significant differences, this may be explained by the limited number of studies. All these factors could increase heterogeneity, and may limit comparability, as similarly discussed in other reviews.[21, 39]

#### Implications of findings for management guidelines

We were able to perform meta-analysis for one of seven risk factors for newborn EOS specified in management guidelines from India.[9, 68] Due to limited data, we were unable to perform meta-analysis for the six other risk factors addressed in the guidelines. Thus, we have discussed them with findings from individual studies as follows:

- Maternal febrile illness (two weeks before delivery): Inconsistent results were reported by studies, possibly due to heterogeneity in defining the timing of maternal febrile illness. One study found peripartum maternal fever and urinary tract infection to be independent risk factors of EOS, only when maternal intra-partum antibiotics were administered.[45] Three studies did not find maternal febrile illness/ infection to significantly increase risk of neonatal sepsis.[43, 50, 56]
- Meconium-stained liquor/ foul-smelling liquor: Studies found meconium stained liquor[45, 47] and chorioamnionitis[50] as independent risk factors of EOS, but not in the absence of intrapartum antibiotics.[45] Foul-smelling liquor was not found to significantly increase the risk of neonatal sepsis, including EOS.[43, 45, 50]
- Prolonged rupture of membranes >24 hours: One study found this to be a risk factor in the pre-intrapartum antibiotics era, but not when intrapartum antibiotics were administered.[45]
- Multiple per-vaginal examinations in labour: Three studies found >3 per-vaginal examinations to independently increase the risk of EOS.[45, 47, 50] There was heterogeneity in the timing of the per-vaginal examinations.
- Prolonged and difficult delivery: Two studies assessing prolonged labour did not find it be a risk factor for EOS (among preterm neonates)[50] or fungal sepsis.[43]
- Perinatal asphyxia: One study assessing APGAR score ≤ 4 at 5 minutes did not find it to significantly increase the risk of EOS.[50]

For LOS, the need for artificial ventilation has been suggested as risk factor in management guidelines, supported by our meta-analysis and individual studies.[9, 63, 68, 69] Due to a limited number of included studies exploring other factors in our review (e.g. invasive procedure, parenteral therapy, NICU stay, poor hygiene/ umbilical cord care, pre-lacteal/ bottle feeding), we were unable to provide conclusive evidence of LOS. Evidence on risk factors, which are usually nosocomial or community-acquired in LOS, is required as a recent population-based study from rural India found 94% of the culture-proven sepsis cases to be of late-onset origin.[70]

Future robust analytical studies with a focus on other neonatal systemic infections (e.g. pneumonia, meningitis) and on community-acquired/ late-onset sepsis are required. Additionally, more systematic reviews and meta-analyses are required in order to better understand if and how clinical sepsis influences the risk factor estimates, and thus may have important implications for informing diagnostic guidelines in India.

### Strengths and limitations

The broad search strategy (designed to favour sensitivity over specificity) and the combination of global and regional databases reduced the risk of missing relevant regional studies. The evidence in this review is derived from studies conducted in tertiary hospital settings, predominantly from urban settings. This limits the generalizability of the review findings. Additionally, this aspect requires caution to be exercised in interpreting and generalizing outborn admissions as a risk factor, due to lack of data on neonates with sepsis in the community and in rural facilities (i.e. neonates who missed getting admitted to urban tertiary healthcare centres, and/or when only inborn neonates were included in the study). A few studies were excluded because of non-response from the authors on crucial questions. Due to low specificity of clinical features, data/studies reporting exclusively on clinical/probable sepsis were excluded. The absence of exposure definitions (e.g. timing of maternal fever, duration of PROM) prevented the inclusion of several studies in our meta-analysis.

## Conclusion

Our meta-analysis found three neonatal (male gender, out born admissions, need for artificial ventilation) and two maternal (gestational age <37 weeks and PROM) factors to significantly increase the risk for sepsis among neonates. Evidence on other important risk factors of neonatal sepsis from India, including for community-acquired and neonatal systemic infections other than neonatal sepsis, is lacking. Robust research and improved reporting on risk factors is required from India, which has the highest global incidence of neonatal sepsis, for improved preventive efforts to reduce the burden of neonatal sepsis in India.

## Supporting information

S1 PRISMA Checklist Completed PRISMA checklist for the study. (DOC)

S1 File Protocol of the systematic review. (DOCX)

S2 File Sensitivity analysis. (DOCX)

S1 Table Definitions and diagnostic criteria in included studies. (DOCX)

S2 Table Risk factor profile of the included studies. (DOCX)

S3 Table Results from studies not included in meta-analysis. (DOCX)

S4 Table Quality assessment of included studies. (DOCX)

S1 Forest Plots Forest plots illustrating subgroup analysis. (DOCX)

S1 Funnel Plots Funnel plots illustrating publication bias assessment. (DOCX)
