# Supplementary material for: Risk factors of neonatal sepsis in India: A systematic review and meta-analysis
Source: PLoS One. 2019 Apr 25;14(4):e0215683. doi: 10.1371/journal.pone.0215683 (PMC6483350; doi:10.1371/journal.pone.0215683)
Supplement: S1 File — (DOCX) [file pone.0215683.s002.docx]

**S1 File**

**Review Protocol**

**Risk factors of neonatal sepsis in India: a protocol for a systematic review and planned meta-analysis**

Shruti Murthy, Myron Anthony Godinho, Vasudeva Guddattu, Leslie Edward Simon Lewis, N. Sreekumaran Nair

**Objective:** We aimed to conduct a systematic review and meta-analysis to identify and assess the evidence on risk factors of neonatal sepsis in India.

**Methods**

The protocol for this review was drafted in accordance with ‘Preferred Reporting Items for Systematic reviews and Meta-analysis Protocols (PRISMA-P) [1] and the “Meta-analysis of observational studies in epidemiology” (MOOSE) guidelines. [2].

**Registration**: Registered on PROSPERO (PROSPERO CRD42017053721).

**Searches**

The following electronic databases will be searched from their inception: PubMed, CINAHL (EBSCOHost), Scopus, Web of Science (Clarivate Analytics), Popline, IndMed, Indian Science Abstracts and Google Scholar. Studies on the first 10 pages of results from Google Scholar from the year 2000 onwards will be screened. Additionally, the reference lists of included studies and systematic reviews will be screened to include potentially relevant studies. Subject experts and researchers will also be contacted to identify any existing relevant studies.

We developed a broad and sensitive search strategy by following the methodology recommended by the Centre for Reviews and Dissemination’s (CRD) ‘Guidance for undertaking reviews in healthcare', literature review and in consultation with subject experts and information scientist. We included free text words and database specific subject headings. The key domains for developing the search strategy included 'neonate', sepsis' and 'risk factor’. The intra-domain search terms were combined using ‘OR’, and inter-domain terms using ‘AND’. Database-specific filters were used to limit the search to ‘Humans’ and ‘India’. If a filter for “India” was unavailable, this was added to the search strategy as the fourth domain, and combined using ‘AND’ with the other three domains. The search strategy for PubMed has been provided on page 6 of this protocol.

**Eligibility criteria**

Type of participants: Neonates were defined as per that given by the World Health Organization (WHO) as infants under 28 days of age. This could include “under/ up to 28 days of age”, “0 to 28 days”.[3] However, it was not a requisite that studies state the age group for inclusion in our review. We thus included studies which specified the population as “neonates” or “newborn”. If “infants” or “under-five” or “child/ children” was specified, we included the study only if the study included a note/section/ sub group on neonatal age group.

Type of disease: Studies conducted on neonatal sepsis were considered for our review, which included the following systemic infections: neonatal septicaemia/sepsis, pneumonia, meningitis, osteomyelitis, arthritis and urinary tract infections.[4]

*Case definition*: There is considerable variation in definitions applied for neonatal sepsis, and a consensus on case definition for neonatal sepsis is still lacking.[4-7]. Using literature review and consultation with neonatologists at referral teaching institution, we decided to include studies using laboratory-dependent diagnostic criteria (e.g. culture, immuno-haematologic, haematologic sepsis parameters) in our review. In addition, to diagnose neonatal pneumonia, studies should use radiological investigations to confirm it, to be eligible for inclusion in our review. [4, 8] Data/ studies which reported using only clinical criteria for diagnosis of neonatal sepsis will be excluded, since clinical signs are not specific to diagnose neonatal sepsis. The case definitions used in included studies will be captured and reported.

Type of studies: The following observational analytic study designs will be included: cohort studies, case-control study and analytical cross-sectional studies which report on one or more risk factors of neonatal sepsis. The studies should report on two outcome groups: one with sepsis and one without sepsis. For cohort study, studies can include one or more cohort groups.[9, 10] If the study design is unclear/ poorly reported, but the study reports data on a comparison group, we will designate the study design as either prospective (data collected when neonate was in the neonatal unit) or retrospective (data collected after neonate had been discharged from the neonatal unit).[11] All intervention study designs, reviews, meta-analysis, commentaries and qualitative research will be excluded. Studies published both as full-text or abstract-only will be included. Studies will not be restricted by languages or time of publication.

Type of setting: We will include studies conducted in India, in both community and hospital settings.

Outcome of our review: The outcome of our review was risk factors of neonatal sepsis. Thus, studies which reported on one or more factors associated with sepsis in neonates were eligible for inclusion.

To be included in our meta-analysis, at least two studies had to report quantitative data for that outcome. This could be either as crude numbers (events and non-events), odds ratio (OR; unadjusted or adjusted), or mean/median along with range/inter-quartile range/standard deviation (SD).

**Study Selection**

Studies will be selected using eligibility criteria by two authors (SM and MG) independently in three stages of title, abstract and full-text screening on EndNote x7.8. During title screening, only those titles that are rejected by both authors will be excluded from our review. Similar process will be followed for abstract screening. For full-text study selection, both authors have to approve the record for inclusion in the review. We will provide the primary reason for exclusion of records in the full text stage. We will outline and summarise the study selection process using a PRISMA chart.[12]

**Data Extraction**

The data extraction form will be developed with inputs from subject and methodological experts, and pilot-tested on one study of each type for completeness on Microsoft Excel 2016. Data on study characteristics and outcome will be abstracted by two authors (SM and MG) independently. We will extract data on study ID, author contact, characteristics of studies, methodology, definitions, type of sepsis, risk factors and outcomes, confounding factors, funding and limitations.

Crude data (dichotomous and continuous) will be extracted for events and non-events where available, and converted to OR [13, 14]. Otherwise, unadjusted and adjusted OR, RR, p-value and CI will be extracted. We will attempt to transform OR where possible from the available data. We will attempt to convert median and range/ interquartile range to mean and SD [14, 15].

Missing data: We will contact authors of studies in an attempt to obtain missing information or gain clarity of information on methodology (e.g. study setting, case definition) and outcomes. In cases where we do not receive the authors’ reply or the reply is inadequate (i.e. retrieval of missing data is not possible), we will exclude that study/ outcome from the review.

**Quality assessment**

Two authors (SM and MG) will independently performed quality assessment of included studies using the Newcastle-Ottawa Scale (NOS) for case-control studies [16], and the National Heart, Lung, and Blood Institute’s (NHLBI) “Quality Assessment Tool for Observational Cohort and Cross-Sectional Studies”.[17] The outcome of this appraisal will be provided in results and also discussed in our review.

Discrepancies, when they arise, will be resolved by discussion and consensus in the presence of senior authors (LL and VG) for all the above procedures.

**Data analysis and reporting**

Data for meta-analysis will be entered on MS Excel by SM and verified by MG. Meta-analysis will be performed using RevMan v. 5.3 [18] by SM and verified by VG. We will use the inverse-variance method and log OR for dichotomous outcomes, and SMD for continuous outcomes.

Heterogeneity will be assessed using Chi^2^ test, I^2^ statistic and Tau^2^, and an I² value of 25-50% will be considered as low, 50-75% as moderate and ≥75% as high heterogeneity.[19] We anticipate substantial heterogeneity in definitions used for sepsis and risk factors, and methods used. We will, therefore, use a random-effects model for meta-analysis [20, 21]. Pooled effect estimates will be reported with 95% confidence intervals (CI). Forest plots will be used to display the results graphically. [22] Estimates will not be pooled across the time of onset of sepsis (i.e. early vs late onset) or type of systemic infection (e.g. sepsis vs pneumonia vs meningitis).

Tables and textual description will be provided for factors for which a meta-analysis will not be possible. The characteristics of studies, risk factor profile of included studies, summary of findings of risk factors not included in meta-analysis and quality assessment will be outlined in tables, along with a concise textual reporting.

**Subgroup analysis**: It is planned to conduct subgroup analyses according to (a) case definitions for sepsis (culture-dependant vs hematologic sepsis parameters), (b) study design (c) type of systemic infection and (d) setting (hospital-based vs community-based). Subgroup analysis will be performed when there are ≥2 studies for each subgroup. The overall results of subgroup analysis for the respective risk factor, will be stated in our review.

**Sensitivity analysis**: To assess the robustness of the pooled effect size, a sensitivity analysis by excluding one study at a time on OpenMeta-analyst [23]. The sources of bias and other study limitations that should be considered when interpreting the findings of the review will be discussed.

**Publication bias:** Publication bias will be assessed if there at least 10 studies in the meta-analysis, using a funnel plot. Egger’s test will be used to assess the degree of asymmetry.[24]

The findings of this systematic review and meta-analysis will be reported in accordance with the ‘Preferred Reporting Items for Systematic reviews and Meta-analysis (PRISMA) [25] and the “Meta-analysis of observational studies in epidemiology” (MOOSE) Guidelines. [2]

**Acknowledgments:** We thank Mrs. Ratheebhai V., Senior Librarian and Information Scientist, Manipal School at Communication, Manipal University, Manipal for her assistance in developing and testing the review search strategy. We thank Dr. Manoj Das, Director-Projects, The INCLEN Trust International, New Delhi, (also a Doctoral Advisory Committee member on SM’s PhD) for his review of and recommendations on this review protocol. We are also grateful to Prof. B. Unnikrishnan, Associate Dean, Kasturba Medical College, Mangaluru, Karnataka, India for his inputs on this protocol.

**Author roles:**

Shruti Murthy: Study design, drafting protocol, data collection, management, analysis and interpretation, drafting protocol and manuscript

Myron A Godinho: Study design, drafting protocol, data collection, management, analysis and interpretation, and manuscript revision

Vasudeva Guddattu: Study design, revising protocol, data analysis and interpretation, manuscript revision, supervision

Leslie E S Lewis: Study design, revising protocol, data validation and interpretation, manuscript revision, supervision

N. Sreekumaran Nair: Study design, revising protocol, data interpretation, manuscript revision, supervision

**Conflict of Interest**: None

**References**

1. Moher D, Shamseer L, Clarke M, Ghersi D, Liberati A, Petticrew M, et al. Preferred reporting items for systematic review and meta-analysis protocols (PRISMA-P) 2015 statement. Systematic reviews. 2015;4(1):1.

2. Stroup DF, Berlin JA, Morton SC, Olkin I, Williamson GD, Rennie D, et al. Meta-analysis of observational studies in epidemiology: a proposal for reporting. Jama. 2000;283(15):2008-12.

3. World Health Organization. Health Topics. Infant, Newborn. 2018. Available from: <http://www.who.int/>.

4. Aggarwal R, Sarkar N, Deorari AK, Paul VK. Sepsis in the newborn. Indian Journal of Pediatrics. 2001;68(12):1143-7.

5. Schlapbach LJ, Kissoon N. Defining pediatric sepsis. JAMA pediatrics. 2018;172(4):312-4.

6. Wynn JL. Defining neonatal sepsis. Current opinion in pediatrics. 2016;28(2):135.

7. Zea-Vera A, Ochoa TJ. Challenges in the diagnosis and management of neonatal sepsis. Journal of Tropical Pediatrics. 2015;61(1):1-13. doi: 10.1093/tropej/fmu079.

8. Duke T. Neonatal pneumonia in developing countries. Archives of Disease in Childhood - Fetal and Neonatal Edition. 2005;90(3):F211-FF9. doi: 10.1136/adc.2003.048108.

9. Dekkers OM, Egger M, Altman DG, Vandenbroucke JP. Distinguishing case series from cohort studies. Annals of internal medicine. 2012;156(1_Part_1):37-40.

10. Mathes T, Pieper D. Clarifying the distinction between case series and cohort studies in systematic reviews of comparative studies: potential impact on body of evidence and workload. BMC medical research methodology. 2017;17(1):107.

11. Women's NCCf, Health Cs. Antibiotics for early-onset neonatal infection: antibiotics for the prevention and treatment of early-onset neonatal infection. 2012.

12. Liberati A, Altman DG, Tetzlaff J, Mulrow C, Gøtzsche PC, Ioannidis JP, et al. The PRISMA statement for reporting systematic reviews and meta-analyses of studies that evaluate health care interventions: explanation and elaboration. Annals of internal medicine. 2009;151(4):W-65-W-94.

13. Chinn S. A simple method for converting an odds ratio to effect size for use in meta‐analysis. Statistics in medicine. 2000;19(22):3127-31.

14. Higgins J, Green S. Cochrane handbook for systematic reviews of interventions. Version 5.1. 0 [updated March 2011]. The Cochrane Collaboration. www cochrane-handbook org. 2011.

15. Wan X, Wang W, Liu J, Tong T. Estimating the sample mean and standard deviation from the sample size, median, range and/or interquartile range. BMC medical research methodology. 2014;14(1):135.

16. Wells G, Shea B, O’connell D, Peterson J, Welch V, Losos M, et al. The Newcastle-Ottawa Scale (NOS) for assessing the quality of nonrandomised studies in meta-analyses. 2000.

17. NIH National Heart Lung and Blood Institute. Study Quality Assessment Tools-Quality Assessment Tool for Observational Cohort and Cross-Sectional Studies. Available from: <https://www.nhlbi.nih.gov/>.

18. Review Manager 2014. Nordic Cochrane Centre, The Cochrane Collaboration. Review Manager 5 (RevMan 5). Version 5.3. Copenhagen: Nordic Cochrane Centre, The Cochrane Collaboration, 2014.

19. Higgins JPT, Thompson SG, Deeks JJ, Altman DG. Measuring inconsistency in meta-analyses. BMJ. 2003;327(7414):557-60. doi: 10.1136/bmj.327.7414.557.

20. DerSimonian R, Laird N. Meta-analysis in clinical trials. Controlled clinical trials. 1986;7(3):177-88.

21. Sonego M, Pellegrin MC, Becker G, Lazzerini M. Risk factors for mortality from acute lower respiratory infections (ALRI) in children under five years of age in low and middle-income countries: a systematic review and meta-analysis of observational studies. PloS one. 2015;10(1):e0116380.

22. DerSimonian R, Laird N. Meta-analysis in clinical trials revisited. Contemporary clinical trials. 2015;45:139-45.

23. Wallace BC, Dahabreh IJ, Trikalinos TA, Lau J, Trow P, Schmid CH. Closing the Gap between Methodologists and End-Users: R as a Computational Back-End. 2012. 2012;49(5):15. Epub 2012-06-30. doi: 10.18637/jss.v049.i05.

24. Egger M, Smith GD, Schneider M, Minder C. Bias in meta-analysis detected by a simple, graphical test. BMJ. 1997;315(7109):629-34.

25. Moher D, Liberati A, Tetzlaff J, Altman DG. Preferred reporting items for systematic reviews and meta-analyses: the PRISMA statement. BMJ. 2009;339. doi: 10.1136/bmj.b2535.

**Search strategy for PubMed**

1. (((((((((("sepsis"[MeSH Major Topic]) OR sepsis[Text Word]) OR septicaemia[Text Word]) OR septicemia[Text Word])) OR ((("pneumonia"[MeSH Major Topic]) OR pneumonia[Text Word]) OR pneumoni*[Text Word])) OR ((((("meningitis"[MeSH Major Topic]) OR meningitis[Text Word]) OR meningoencephalitis[Text Word]) OR encephalitis[Text Word]) OR "encephalitis"[MeSH Major Topic])) OR ((((("arthritis"[MeSH Major Topic]) OR bone infection[Text Word]) OR arthritis[Text Word]) OR "osteomyelitis"[MeSH Major Topic]) OR osteomyelitis[Text Word])) OR (((((((((((((((("urinary tract infections"[MeSH Major Topic]) OR "urinary tract infection"[Text Word]) OR "urethritis"[Text Word]) OR "cystitis"[Text Word]) OR "bacteriuria"[Text Word]) OR "bacteremia"[Text Word]) OR pyogen*[Text Word]) OR "epididymitis"[Text Word]) OR "prostatits"[Text Word]) OR "vesicoureteral reflux"[Text Word]) OR "pyuria"[Text Word]) OR "trigonitis"[Text Word]) OR "pyelonephritis"[Text Word]) OR "pyonephrosis"[Text Word]) OR "hydronephrosis"[Text Word]) OR "urinary infection"[Text Word])) OR (((((("lung infection"[Text Word]) OR "respiratory tract infections"[MeSH Major Topic]) OR "respiratory tract infections"[Text Word]) OR "respiratory infection"[Text Word]) OR "blood infection"[Text Word]) OR "brain infection"[Text Word])) OR "joint infection"[Text Word]

2. (((((((((("infant, newborn"[MeSH Major Topic]) OR "neonatology"[MeSH Major Topic]) OR "infant"[Text Word]) OR "newborn"[Text Word]) OR "neonate"[Text Word]) OR "neonatal"[Text Word]) OR "toddler"[Text Word]) OR "baby"[Text Word]) OR "babies"[Text Word]) OR "paediatric"[Text Word]) OR "paediatric"[Text Word]

3. ((((((((((("risk"[MeSH Major Topic]) OR "risk factors"[MeSH Major Topic]) OR "association"[MeSH Major Topic]) OR "causality"[MeSH Major Topic]) OR "risk"[Text Word]) OR "risk factor"[Text Word]) OR "determinant"[Text Word]) OR "predictor"[Text Word]) OR "causal"[Text Word]) OR "association"[Text Word]) OR "causal factor"[Text Word]) OR "odds ratio"[MeSH Major Topic]

4. (((((((((((((((((((((((((((((((((((((((((((((((((("india"[MeSH Terms]) OR "india") OR "indian") OR "tamil nadu") OR "andhra pradesh") OR "kerala") OR "goa") OR "karnataka") OR "maharashtra") OR "gujarat") OR "rajasthan") OR "delhi") OR "national capital region") OR "delhi ncr") OR "chandigarh") OR "punjab") OR "jammu") OR "kashmir") OR "himachal pradesh") OR "uttarakhand") OR "uttaranchal") OR "uttar pradesh") OR "madhya pradesh") OR "jharkhand") OR "chhattisgarh") OR "bihar") OR "orissa") OR "odisha") OR "telangana") OR "west bengal") OR "sikkim") OR "assam") OR "nagaland") OR "manipur") OR "meghalaya") OR "tripura") OR "mizoram") OR "arunachal pradesh") OR "dadra") OR "nagar haveli") OR "daman") OR "diu") OR "andaman") OR "nicobar") OR "lakshadweep") OR "haryana") OR "puducherry") OR "pondicherry") OR ("andaman and nicobar")) OR ("daman and diu")) OR ("dadra and nagar haveli")

5. #1 AND #2 AND #3 AND #4

Filters: Humans
