# Supplementary material for: Risk factors of neonatal sepsis in India: A systematic review and meta-analysis
Source: PLoS One. 2019 Apr 25;14(4):e0215683. doi: 10.1371/journal.pone.0215683 (PMC6483350; doi:10.1371/journal.pone.0215683)

# **S2 File**

# **Sensitivity analysis** (Leave-one-out meta-analysis)

# The forest plots below illustrate the sensitivity analysis for maternal and neonatal risk factors of neonatal sepsis. On the forest plots, the red dotted line and the blue diamond correspond to the pooled effect estimate from random-effects meta-analysis. The black squares corresponding to each study represent the change in the pooled effect estimate on exclusion of that study. The horizontal lines across the blue diamond/ black squares represent the confidence intervals. The forest plots are supplemented with textual descriptions.

# *Neonatal factors*

**1. Male gender**

On conducting the leave-one-out meta-analysis, the minimum pooled OR obtained was 1.105 (95% CI: 0.98, 1.24; Tapader2014 [48]) and the maximum pooled OR was 1.47 (95% CI: 1.04, 2.07; DeNIS2016 [47]). Exclusion of either of the three studies viz Chaurasia2015 [48], Prashant2013 [57] or Tapader2014 [48] resulted in the loss of significance with pooled effect estimates of 1.29 (95% CI: 0.99, 1.66), 1.29 (95% CI: 0.99, 1.69) and 1.1 (95% CI: 0.98, 1.24) respectively.


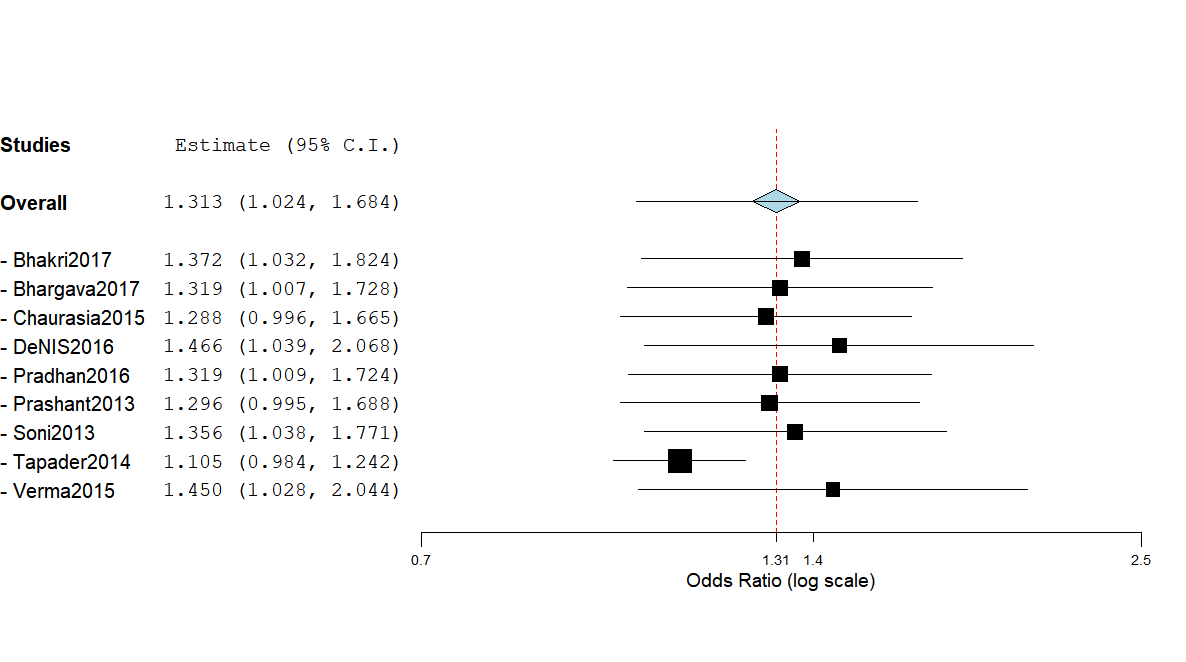


**2. Low birth weight (<2500 grams)**

Leave-one-out meta-analysis did not result in a significant difference in pooled estimate results, with a resultant minimum pooled OR of 1.25, which also resulted in the least uncertainty (95% CI: 0.78, 2.02) when Sundaram2009 [58] was excluded. The maximum pooled effect estimate was 3.09 (95% CI: 0.54, 17.65; Bhakri2017 [51]). Though there was no marked difference in the pooled estimate, there was greater uncertainly when DeNIS2016 [47] was excluded.


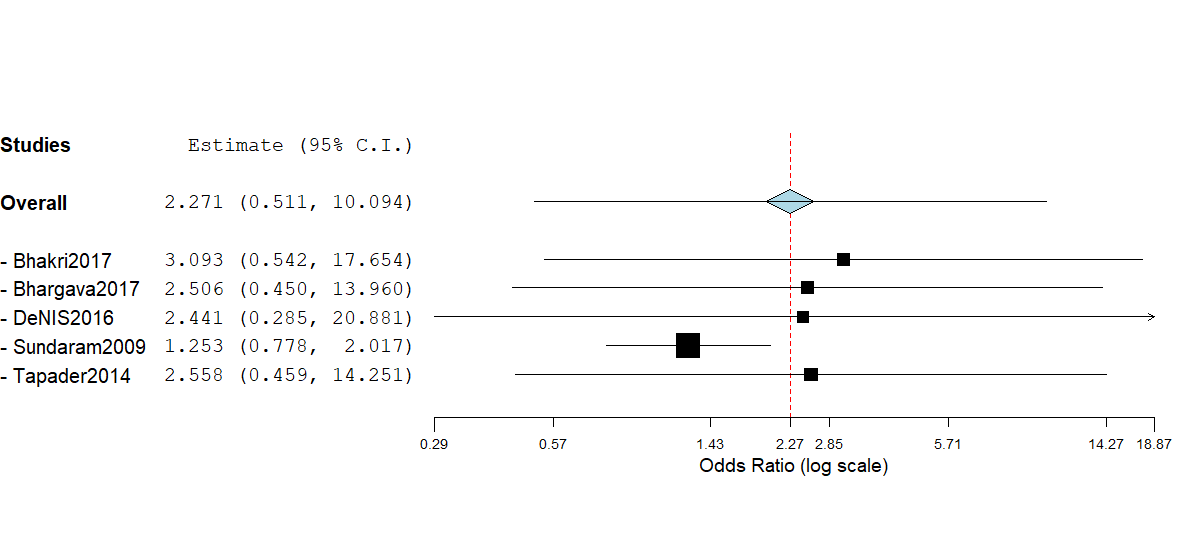


**3. Birthweight (continuous)**

Leave-one-out meta-analysis revealed that the exclusion of one study i.e. Prashant2013 [57] resulted in a pooled effect estimate of -0.74 (95% CI: -1.5, 0.03) and thus resulted in the loss of significance. On performing the leave-one-out meta-analysis, the maximum pooled effect estimate obtained was -0.65 (95% CI: -1.22, -0.08; Soni2013 [54]) and the minimum pooled effect estimate was -1.02 (95% CI: -1.33, -0.71; Pradhan2016 [53]).


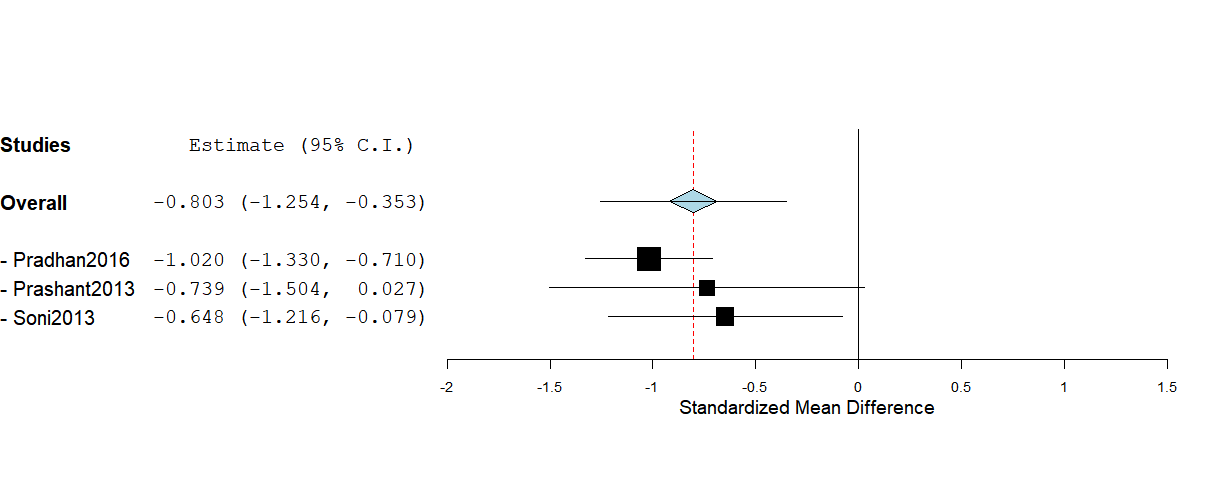


**4. Outborn admission**

On conducting a leave-one-out meta-analysis, exclusion of Bhakri2017 [51] resulted in an increase in the pooled effect estimate to 8.95 (95% CI: 3.03, 26.42). Excluding Chaurasia2015 [43] did not result in a marked difference in the pooled effect estimate but resulted in a greater uncertainty (95% CI: 1.17, 23.10). The minimum pooled effect estimate changed to 4.45 (95% CI: 1.85, 11.14) after the leave-one-out meta-analysis.


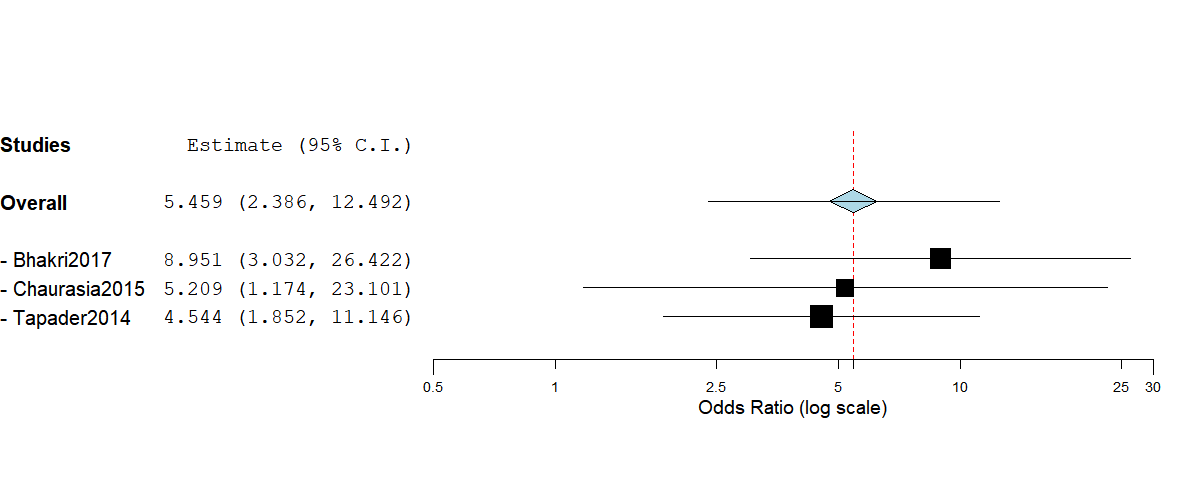


**5. Need for artificial ventilation**

The maximum pooled effect estimate obtained after leave-one-out meta-analysis was 22.87, also resulting in a greater uncertainty (95% CI: 8.17, 63.99) when Chaurasia2015 [43] was excluded. The minimum pooled effect estimate obtained was 15.8 (95% 4.86, 51.43) when the largest study (Bhargava2017 [56]) was excluded.


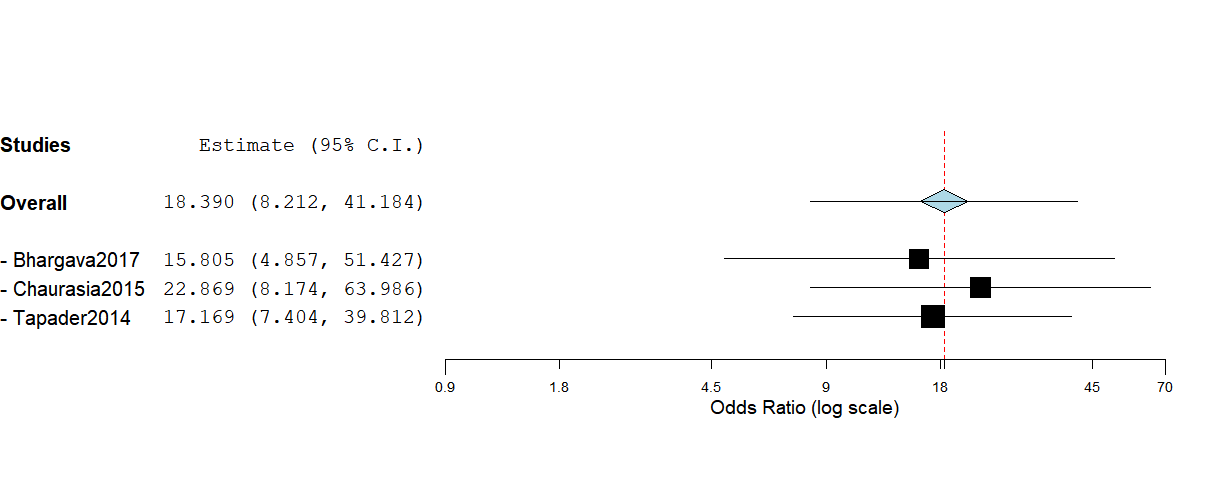


## *Maternal factors*

**1. Delivery at <37 weeks of gestation**

There were no marked differences in the pooled effect estimate on performing the sensitivity analysis. The minimum and maximum pooled effect estimates were 1.81 (95% CI: 1.15, 2.83; Verma2015 [55]) and 2.39 (95% CI: 1.68, 3.39; Bhargava2017 [56]) respectively after the leave-one-out meta-analysis.


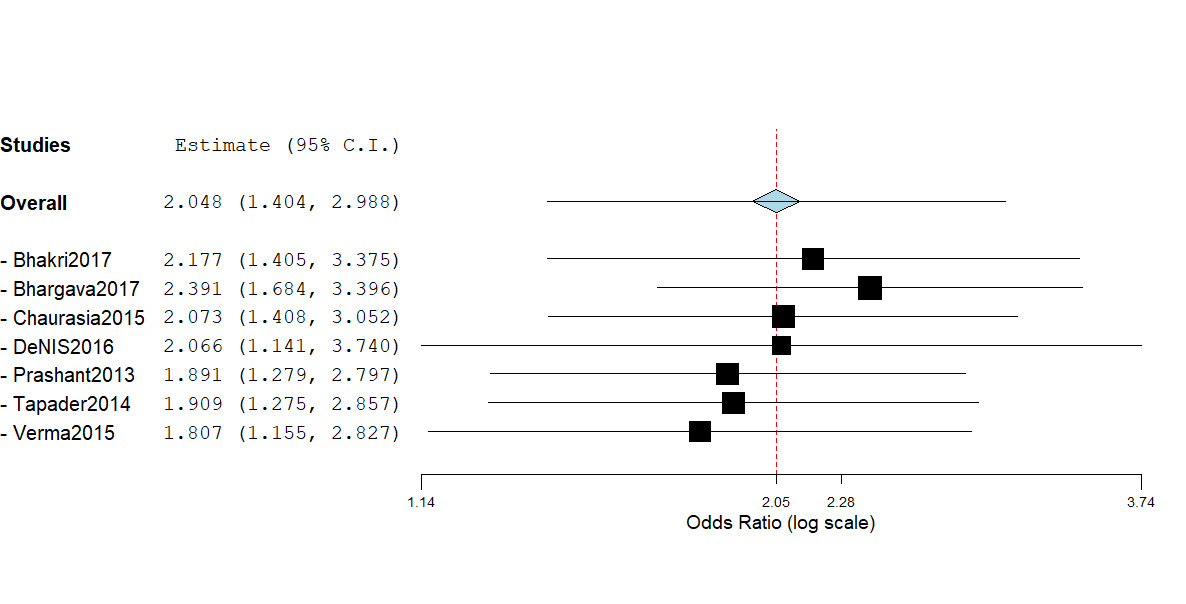


**2. Vaginal delivery**

Leave-one-out meta-analysis did not show marked differences in the results. The minimum and maximum pooled effect estimates were 1.29 (95% CI: 0.54, 3.09; Tapader2014 [48]) and 3.01 (95% CI: 0.91, 10.44; Bhargava2017 [56]) respectively.


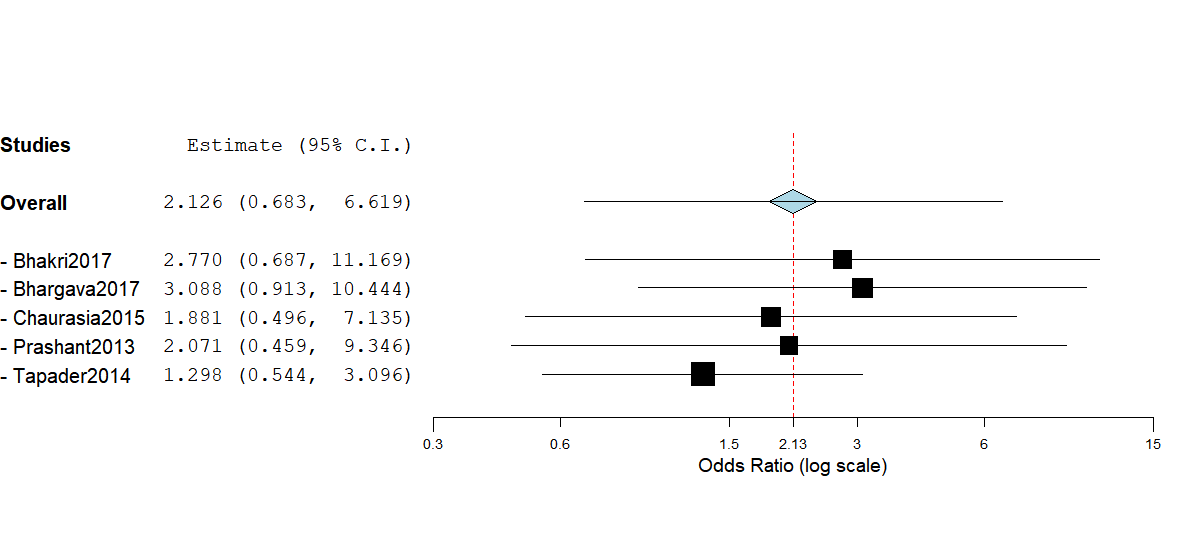


**3. Premature rupture of membranes**

The minimum pooled effect estimate, after leave-one-out meta-analysis, was 10.02 (95% CI: 4.8, 20.93) when Chaurasia2015 [43] was excluded. On excluding the largest study i.e. Bhakri2017 [51], the pooled effect estimate increased to 27.32 and had the greatest uncertainty (95% CI: 4.82, 154.83).


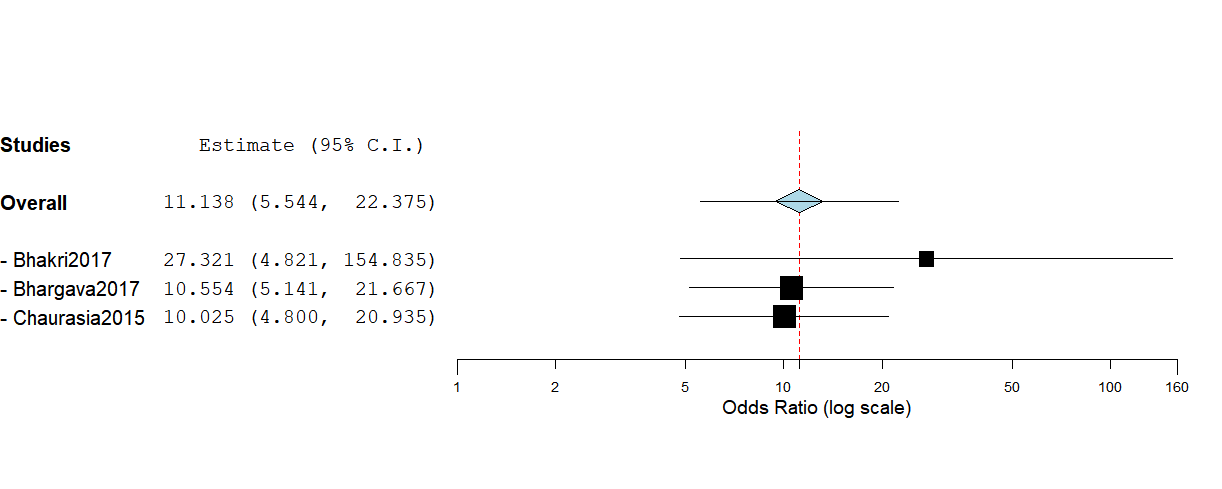

Supplement: S2 File — (DOCX) [file pone.0215683.s003.docx]
