# Supplementary material for: Risk factors of neonatal sepsis in India: A systematic review and meta-analysis
Source: PLoS One. 2019 Apr 25;14(4):e0215683. doi: 10.1371/journal.pone.0215683 (PMC6483350; doi:10.1371/journal.pone.0215683)
Supplement: S1 Table — (DOCX) [file pone.0215683.s004.docx]

**S1 Table**

# **Definitions in the included studies**

**Table S1A: Definitions, eligibility criteria and guidelines**

| **Sl. No** | **Study ID** | **Definition of EOS and LOS** | **Guideline used** | **Inclusion criteria** | **Exclusion criteria** | | **Primary aim to determine risk factor?** |
| --- | --- | --- | --- | --- | --- | --- | --- |
| **Neonatal sepsis (diagnosis using haematologic sepsis parameters)** | | | | | | | |
|  | Bhakri 2017 | - | - | Cases- neonatal sepsis  Controls- matched healthy neonates | Thrombocytopenia (congenital and acquired causes) | | No |
|  | Das 2016 | - | - | Case: Term infants > 37 weeks gestation, with sepsis (clinical + laboratory) at the study site  Control: healthy, matched (age and sex)  Parent consent | From twins or higher multiples pregnancies, autoimmune diseases, birth defects | | No |
|  | DeNIS 2016a,b | EO: ≤72 hours of life  LO: >72 to 28 days of life | CDC NHSN criteria, Young Infant Study Algorithm | All neonates admitted to NICUs of study sites, consent | Inborn neonates who were recruited in a concurrent trial | | Yes |
|  | Pradhan 2016 | - | European Medicines Agency | Neonates with suspected sepsis, and healthy neonates undergoing routine screening for congenital hemoglobinopathies, no suspicion of sepsis  Consent from legal representatives | Neonates with suspected sepsis receiving G-CSF therapy | | No |
|  | Soni 2013 | - | - | Cases: Consecutive neonates with sepsis signs & symptoms at NICU  Controls: neonates with hyperbilirubinemia admitted for phototherapy  Parent consent | Congenital disorders, immune deficiencies | | No |
|  | Verma 2015 | - | - | inborn babies at NICU with clinical sepsis | - | | No |
| **Neonatal sepsis (culture-positive)** | | | | | | | |
|  | Bhargava 2017 | - | CDC,  CLSI | Case: neonates with sepsis confirmed by CDC criteria, admitted to the ward.  Control: neonates without sepsis, admitted to same ward on same day (+ or - 2 days) | NS | | Yes |
|  | Chaurasia 2015  fungal sepsis | - | NCCLS* | Case: neonate in NICU, candida isolated in blood, BW <2kg, parent consent  Control: Matched (GA, BW, sex), neonate in NICU, blood culture negative for candida. | NS | | Yes |
|  | DeNIS 2016a,b | EO: ≤72 hours of life  LO: >72 to 28 days of life | CDC NHSN criteria, Young Infant Study Algorithm | All neonates admitted to NICUs of study sites, consent | Inborn neonates who were recruited in a concurrent trial | | Yes |
|  | Dutta 2010  EOS | EO: <72 hours of life | - | Inborn babies, gestational age ≤34 weeks, parent consent | discharged <72 hours of life, major malformations, logistic reasons | | Yes |
|  | Prashant 2013 | EO: within 72 hours of birth  LO: clinical deterioration >3 days of age | CLSI | Neonates (preterm, term) in NICU, clinical signs of sepsis, parent consent | NEC (radiologically determined), on IV antibiotics during sepsis evaluated, congenital disorders, chromosomal abnormalities, inborn errors of metabolism, IU viral infections, recent surgery | | No |
|  | Santhanam 2017  EOGBS | EO: within 72 hours of life |  | Case: babies admitted to study site during study period, with invasive GBS infection <72 hrs of birth  Control: 2 babies born before and after each case |  | | Yes |
|  | Sundaram 2009 | EO: ≤ 72 hours of life  LO: > 72 hours of life | - | all babies born in hospital during study | - | | No |
|  | Tapader 2014 | - | - | E. coli bloodstream isolates from neonates with clinically suspected sepsis from 3 study sites.  Faecal isolates from uncomplicated neonates admitted to postnatal ward of 3^rd^ study site. | - | | No |
| **Neonatal Ventilator-Associated Pneumonia (VAP)** | | | | | | | |
|  | Tripathi 2009 | EO: <5days of MV  LO: ≥ 5 days after MV | CDC, NNIS | Intubated neonates in NICU during study period | Neonates requiring MV<48 hours, neonates with pneumonia at MV initiation | | Yes |
|  | Vijayakanthi 2015 | - | CDC | Neonates on MV > 48 hours | - | | Yes |
| **Neonatal meningitis** | | | | | | | |
| 1. | DeNIS 2016b | EO: ≤72 hours of life  LO: >72 to 28 days of life | CDC NHSN criteria, Young Infant Study Algorithm | All neonates admitted to NICUs of study sites, consent | Inborn neonates who were recruited in a concurrent trial | Yes | |

C- Clinical, BC: Blood culture, SS: Sepsis screen, R: Radiology, I: Imaging, RF- Risk factors, T- appropriate treatment initiated

^- culture taken from site appropriate for type of sepsis

NCCLS: National Committee for Clinical Quality Control; CLSI: Clinical and Laboratory Standards Institute

**TableS1B: Diagnostic criteria and guidelines used in included studies**

| **Sl. No** | **Study ID** | **Criteria for case definition** | **Clinical** | **Septic Screen/ laboratory** | **Culture** | **Other investigations** |
| --- | --- | --- | --- | --- | --- | --- |
| **Neonatal sepsis (diagnosis using haematologic sepsis parameters)** | | | | | | |
|  | Bhakri 2017 | C+SS/BC | lethargy, decreased feeding, fever, hypothermia, vomiting, difficult  breathing, apnoea, bulging fontanels, abdominal distension, multiple>10 skin pustules | CRP, TLC, µESR, ITR, Platelet count, MPV, PDW,  Also-DLC, PBF | Blood (CSF/urine- as required) | - |
|  | Das 2016 | laboratory | Temperature instability, respiratory distress, convulsions, jaundice, feeding problems, autonomic disturbances | Others: 25(OH)D serum level, Vitamin D Receptor polymorphisms | - | - |
|  | DeNIS 2016a,b | CDC-NHSN and Young Infant Study Algorithm  CNS: RF/C+ SS/BC+T | Any of the signs/symptoms or maternal risk factors was considered. | (for CNS) included any 2 of: TLC, ANC, ITR, CRP, µESR (cut-off values specified). | Blood | Radiological evidence for pneumonia |
|  |  |  | Please refer the detailed list/criteria and working case definitions provided in the study, adapted from CDC-NHSN and Young Infant Study Algorithm for CPS, CNS, fungal sepsis, meningitis and urinary tract infection. | | | |
|  | Pradhan 2016 | C+SS/BC | At least 2 clinical criteria from the list. | Suspected: CRP, TLC, ITR, PC, base excess, random glucose  Healthy- CRP, TLC, PC  (≥2 of above to confirm CNS, cut-off values specified)  Others: CSF analysis, nCD64, mHLA-DR | Blood  Other sites, as indicated | Chest and abdominal X-ray where indicated |
|  |  |  | Please refer study for detailed criteria and EMA guidelines for working case definition of sepsis. | | | |
|  | Soni 2013 | C+SS | - | TLC, ANC, ITR, µESR, CRP (≥2 of above to confirm sepsis; cut-off values specified)  Also-mCD64, nCD64 | - | Blood, CSF/endotracheal culture  (culture not requisite to confirm sepsis) |
|  | Verma 2015 | C+SS/BC | Fever, abdominal distension, refusal to feed, vomiting, excessive cry, cold extremities, pallor, cyanosis, lethargy, bleeding, grunting, respiratory distress, apnoea, jaundice, diarrhoea, seizure, bulging anterior fontanelle, umbilical discharge, oral candidiasis, rash, circumoral hue, others, pyoderma, sclerema | TLC, ITR, CRP, µESR band cell count  (≥2 of above to confirm sepsis; cut-off values specified) | Blood | - |
| **Neonatal sepsis (culture-positive)** | | | | | | |
|  | Bhargava 2017 | C | - | - | Blood | - |
|  | Chaurasia 2015  Fungal sepsis | C/SS+BC | Temperature instability, abdominal distension, increased respiratory efforts or apnoea, gastric residual | TLC, CRP, Platelet count | Blood | - |
|  | DeNIS 2016a,b | CDC-NHSN and Young Infant Study Algorithm  CPS: RF/C+ BC+T | Any of the signs/symptoms or maternal risk factors was considered. | (for CNS) included any 2 of: TLC, ANC, ITR, CRP, µESR (cut-off values specified). | Blood | Radiological evidence for pneumonia |
|  |  |  | Please refer the detailed list/criteria and working case definitions provided in the study, adapted from CDC-NHSN and Young Infant Study Algorithm for CPS, CNS, fungal sepsis, meningitis and urinary tract infection. | | | |
|  | Dutta 2010  EOS | C+BC (or) BC | Not specified | - | Blood | NA |
|  | Prashant 2013 | C/SS/I+BC | Temperature instability, abdominal distension, feeding intolerance, disordered peripheral circulation, irritable, lethargy, hepatosplenomegaly (at least 3 of above)  OR  Respiratory dysfunction (tachypnoea, apnoea, increased FiO_2_) AND circulatory dysfunction (bradycardia, tachycardia, disordered peripheral circulation) | CRP, cytokines (IL-6, IL-8, TNF-α), sCD163 | Blood | Imaging |
|  | Santhanam 2017  EOGBS | C/CSF | - | - | Blood/ CSF | - |
|  | Sundaram 2009 | RF/C+C | Systemic signs e.g. abdominal distension, grunting, apnoea, chest retractions, hypothermia, lethargy, tachycardia, increased pre-feed aspirate | - | Blood | - |
|  | Tapader 2014 | C, SS, BC | (1 or more of) abdominal distention, temperature instability, hypotension, tachypnoea, lethargy, poor feeding, apnoea, poor perfusion, tachycardia. | TLC, ANC, CRP, µESR  (any 2; cut-off values specified) | Blood | - |
| **Neonatal Ventilator-Associated Pneumonia (VAP)** | | | | | | |
|  | Tripathi 2009 | CDC, NNIS (C/L/BC + R) | Fever, chest signs | TLC, DLC | Endotracheal aspirate, blood | Chest X-ray |
|  | Vijayakanthi 2015 | CDC criteria  C+R | Worsening gas exchange, increasing oxygen requirements/ ventilator requirements AND  (at least 3 of) temperature instability, leucocytosis/ leukopenia & left shift, increasing suctioning requirements/ respiratory secretions, tachypnoea, nasal flaring with chest retractions, grunting, wheezing/ rales/ rhonchi, apnoea, tachycardia/ bradycardia | - | Endotracheal aspirate | ≥2 serial chest X-rays (presence of at least 1 of)  new/ progressive persistent infiltrate, cavitation, consolidation, pneumatoceles. |
| **Neonatal meningitis** | | | | | | |
|  | DeNIS 2016b | CDC-NHSN and Young Infant Study Algorithm | At least one of the specified symptoms/ symptoms | CSF protein, CSF white cell, CSF/ blood glucose | CSF | - |
|  |  |  | Please refer the detailed list/criteria and working case definitions provided in the study, adapted from CDC-NHSN and Young Infant Study Algorithm for CPS, CNS, fungal sepsis, meningitis and urinary tract infection. | | | |

C- Clinical, BC: Blood culture, CSF: Cerebrospinal Fluid; SS: Sepsis screen, R: Radiology, I: Imaging, RF- Risk factors, T- appropriate treatment initiated

^- culture taken from site appropriate for type of sepsis; CNS: Culture-Negative Sepsis; CPS: Culture-Positive Sepsis

TNC: Total Neutrophil Count; ANC: Absolute Neutrophil Count; µESR: micro Erythrocyte Sedimentation Rate; MPV: Mean Platelet Volume; PDW: Platelet Distribution Width; PBF: Peripheral Blood Film examination; IL: Interleukin; TNF: Tumour Necrosis Factor; PC: Platelet Count

DLC: Differential Leucocyte Count; TLC: Total Leucocyte Count; CRP: C-Reactive Protein; ITR: Immature to Total neutrophil Ratio
