# Supplementary material for: Risk factors of neonatal sepsis in India: A systematic review and meta-analysis
Source: PLoS One. 2019 Apr 25;14(4):e0215683. doi: 10.1371/journal.pone.0215683 (PMC6483350; doi:10.1371/journal.pone.0215683)
Supplement: S2 Table — (DOCX) [file pone.0215683.s005.docx]

**S2 Table**

# **Table S2A: Risk factor profile of the included studies**

| **Sl. No** | **Study ID** | **Risk factor(s)** | | | **Neonate-related** | **Maternal** |
| --- | --- | --- | --- | --- | --- | --- |
|  |  | **Total** | **N** | **M** |  |  |
| **Neonatal sepsis (diagnosis using haematologic sepsis parameters)** | | | | | | |
|  | Bhakri 2017 | 10 | 5 | 5 | Admission type, sex, birthweight, maturity, resuscitation at birth | Gestational age, mode of delivery, gravida, history of multiple PV exam, prolonged rupture of membranes (ROM)>18 hours |
|  | Das 2016 | 1 | 1 | - | Vitamin D level | - |
|  | DeNIS 2016a,b | 10 | 5 | 5 | Neonatal Sepsis: birthweight  EOS: Sex, birthweight, major malformations  LOS: birthweight, sex, intravenous fluids, mechanical ventilation duration | Neonatal Sepsis: Gestational age  EOS: Gestational age, meconium stained liquor, antenatal visits, mode of delivery, prolonged ROM >18 hours  LOS: Gestational age |
|  | Pradhan 2016 | 5 | 4 | 1 | Age, sex, birthweight, APGAR score at 5 minutes | Gestational age |
|  | Soni 2013 | 4 | 3 | 1 | Age, sex, birthweight | Gestational age |
|  | Verma 2015 | 2 | 1 | 1 | Sex | Gestational age |
| **Neonatal sepsis (culture-positive)** | | | | | | |
|  | Bhargava 2017 | 16 | 10 | 6 | Age, sex, birthweight, resuscitation at birth, mechanical ventilation, venous catheter insertion, expressed/ formula feed, delayed enteral feed, congenital anomalies | Gestational age, mode of delivery, meconium staining, prolonged ROM>18 hours, infection, prior- surgery, steroid use, antibiotic use. |
|  | Chaurasia 2015  Fungal sepsis | 13 | 6 | 7 | Admission type, sex, birthweight, mechanical ventilation, prolonged hospital stay (>7 days), prior antibiotic use (>7 days) | Gestational age, mode of delivery, foul smelling liquor, prolonged labour, prolonged ROM >18 hours, febrile illness, place of living |
|  | DeNIS 2016a,b | 10 | 5 | 5 | Neonatal Sepsis: birthweight  EOS: Sex, birthweight, major malformations  LOS: birthweight, sex, intravenous fluids, mechanical ventilation duration | Neonatal Sepsis: Gestational age  EOS: Gestational age, meconium stained liquor, antenatal visits, mode of delivery, prolonged ROM >18 hours  LOS: Gestational age |
|  | Dutta 2010  EOS | 14 | 4 | 10 | Sex, birthweight, maturity, APGAR score ≤ 4 at 5 minutes | Gestational age, clinical chorioamnionitis, foul smelling liquor, prolonged labor (> 18 hours), spontaneous premature onset of labor, prolonged rupture of membranes (> 18 hours), preterm premature ROM, maternal fever, intrapartum antibiotics, per vaginal exam ≥3 |
|  | Prashant 2013 | 5 | 3 | 2 | Sex, birthweight, APGAR <7 at 1, 5 minutes | Gestational age, mode of delivery |
|  | Santhanam 2017  EOBGS | 14 | 3 | 11 | Birthweight, resuscitation at birth, CTG abnormality | Gestational age, gravida, mode of delivery, urinary tract infection, per vaginal exam >3 after ROM, prolonged ROM (>18, > 24 hours), prelabour ROM, peripartum fever, meconium-stained amniotic fluid, chorioamnionitis |
|  | Sundaram 2008 | 1 | 1 | - | Birthweight | - |
|  | Tapader 2014 | 6 | 4 | 2 | Sex, admission type, birthweight, mechanical ventilation | Gestational age, mode of delivery, others risk factors (unspecified) |
| **Neonatal Ventilator-Associated Pneumonia (VAP)** | | | | | | |
|  | Tripathi 2009 | 10 | 9 | 1 | Age at admission, sex, birthweight, prematurity, SGA/AGA/LGA, mechanical ventilation duration, reintubations, resuscitation at birth, NICU stay length | Premature rupture of membranes |
|  | Vijayakanthi 2015 | 7 | 6 | 1 | Level III stay, unstable cardiopulmonary assessment at admission, respiratory distress at admission, prolonged ventilation, repeated intubations (>1), prolonged hospitalization | Home delivery |
| **Neonatal meningitis** | | | | | | |
|  | DeNIS 2016b | 2 | 1 | 1 | Birth weight | Gestational age |

**Table S2B: Risk factor profile of included studies: Neonate-related Factor**

| **Neonate-related factor** | **Sepsis** | | | **VAP** | **Meningitis** |
| --- | --- | --- | --- | --- | --- |
|  | **All sepsis*** | **EOS** | **LOS** |  |  |
| **No. of studies >10** | | | | | |
| Birthweight | 12 | 3 | 1 | 1 | 1 |
| Sex | 9 | 2 | 1 | 1 |  |
| **No. of studies= 3** | | | | | |
| Admission type | 3 |  |  |  |  |
| Congenital anomalies | 2 | 1 |  |  |  |
| Length of hospitalization | 1 |  |  | 2 |  |
| Mechanical ventilation (MV) | 2 |  |  | 1 |  |
| Duration of MV |  |  | 1 | 2 |  |
| APGAR  < 7 (1,5 min)  ≤ 4 (5 min) | 1  1 | 1 |  |  |  |
| **No. of studies= 2** | | | | | |
| SGA/LGA/AGA |  | 1 |  | 1 |  |
| Resuscitation at birth | 1 |  |  | 1 |  |
| Repeated intubations |  |  |  | 2 |  |
| **No. of studies= 1** | | | | | |
| Age |  |  |  | 1 |  |
| Level III stay |  |  |  | 1 |  |
| At admission:  Respiratory distress  Cardiac abnormalities | 1 |  |  | 1 |  |
| Intravenous fluids |  |  | 1 |  |  |
| Venous catheter insertion | 1 |  |  |  |  |
| Feeding:  Expressed or formula feed  Delayed enteral feed | 1  1 |  |  |  |  |
| Prior antibiotic use > 7 days | 1 |  |  |  |  |
| Vitamin D level | 1 |  |  |  |  |

***** All sepsis is not a sum of EOS+ LOS

**Table S2C: Risk factor profile of included studies: Maternal Factor**

| **Maternal-related factor** | **Sepsis** | | | **VAP** | **Meningitis** |
| --- | --- | --- | --- | --- | --- |
|  | **All sepsis*** | **EOS** | **LOS** |  |  |
| **No. of studies > 10** | | | | | |
| **ROM/ Labour**  Prolonged ROM> 18 hours  > 24 hours  Prelabour ROM  Spontaneous premature onset of labour  Preterm premature ROM  Prolonged labour > 18 hours  Premature rupture of membranes |  |  |  |  |  |
|  | 3  1 | 3  1  1  1  1  1 |  | 1 |  |
| **No. of studies 5 - 10** | | | | | |
| Gestational age at delivery | 9 | 3 | 1 |  | 1 |
| Mode of delivery | 5 | 2 |  |  |  |
| Maternal febrile illness/ infection  Peripartum fever  Maternal fever  Infection  Urinary Tract Infection | 1  1 | 1  1  1 |  |  |  |
| **No. of studies= 3** | | | | | |
| Meconium-stained liquor | 1 | 2 |  |  |  |
| >3 per vaginal examinations after ROM | 1 | 2 |  |  |  |
| **No. of studies= 2** | | | | | |
| Clinical Chorioamnionitis |  | 2 |  |  |  |
| Foul smelling liquor | 1 | 1 |  |  |  |
| **No. of studies= 1** | | | | | |
| Gravida | 1 |  |  |  |  |
| Antibiotics use  Intrapartum antibiotics  Prior surgery | 1  1 | 1 |  |  |  |
| Place of living |  | 1 |  |  |  |
| Place of delivery |  |  |  | 1 |  |
| Antenatal visits |  | 1 |  |  |  |
| Others (not specified) | 1 |  |  |  |  |

***** All sepsis is not a sum of EOS+ LOS
