# Supplementary material for: Risk factors of neonatal sepsis in India: A systematic review and meta-analysis
Source: PLoS One. 2019 Apr 25;14(4):e0215683. doi: 10.1371/journal.pone.0215683 (PMC6483350; doi:10.1371/journal.pone.0215683)
Supplement: S3 Table — (DOCX) [file pone.0215683.s006.docx]

**S3 Table**

# **Results from studies not included in meta-analysis for neonatal sepsis**

**Table S3A: Significant and non-significant factors from studies not included in meta-analysis for neonatal sepsis**

| **Risk factor** | **Studies- significant** | **Studies- Non-significant** |
| --- | --- | --- |
| **NEONATAL** | | |
| Birth weight | - | **Chaurasia 2015**  <1 kg: 1.3 (0.31,5.40), p=0.7  1-1.5: 0.8 (0.21,2.97), p=0.74 |
| Small-for-gestational age | - | **Bhakri 2017**  1.42 (0.72, 2.79), p=0.31 |
| Vitamin D level  (Median ng/ml) | **Das 2016**  p<0.0001  [Sepsis: 12.16 (3.84, 22.22); No sepsis: 30.22 (0.08, 46.78)] | - |
| Expressed/formula feed  Delayed enteral feed | **Bhargava 2017**  GPS: 5 (1.31, 19.07), p=0.02  GNS: 39.32 (2.16, 714.66), p=0.01  **Bhargava 2017** (Gram negative sepsis)  14.48 (3.4, 61.7), p<0.001 | **Bhargava 2017** (Gram positive sepsis)  0.71 (0.14, 3.6), p=0.68 |
| Duration of hospitalization | **Chaurasia 2015** (> 7days)  11.6 (3.39, 40.22), p=0.0001 | - |
| Venous catheter | **Bhargava 2017** (Gram negative sepsis)  24.35 (1.32, 448.52), p=0.03 | **Bhargava 2017** (Gram positive sepsis)  1.67 (0.40, 6.87), p=0.48 |
| Congenital anomalies | **Bhargava 2017** (Gram positive sepsis)  79.95 (4.34, 1473.67), p=0.003 | **Bhargava 2017** (Gram negative sepsis)  0.44 (0.14, 1.38), p=0.16 |
| Prior antibiotics use | **Chaurasia 2015 (**>7 days)  20 (5.38,74.29), p<0.0001 | - |
| **MATERNAL** | | |
| Prolonged labour | - | **Chaurasia 2015**  0.48 (0.04, 5.63), p=0.56 |
| Multiple PV examination | **Bhakri 2017**  9.79 (1.22, 78.81), p=0.03 | - |
| Gravida | - | **Bhakri 2017**  Primigravida: 0.67 (0.38, 1.17), p=0.16 |
| Maternal infection  Febrile illness | - | **Bhargava 2017**  GPS- 0.18 (0.01, 4.04), p=0.28  GNS- 0.31 (0.03, 3.16), p=0.32  **Chaurasia 2015**  1.98 (0.51, 7.63), p=0.32 |
| Foul-smelling liquor | - | **Chaurasia 2015**  4.26 (0.81, 22.53), p=0.09 |
| Place of living | - | **Chaurasia 2015**  2.61 (0.92,7.46), p= 0.07 |
| Obstetric history  antibiotic use  Surgery  Steroid | **Bhargava 2017**  GPS: 33.2, p=0.02  GNS: 5.33 (1.59, 17.83), p=0.005  GPS: 0.18 (0.03, 0.97), p=0.04  GNS: 45.69 (2.52, 829), p=0.009  - | **Bhargava 2017**  GPS: 5.44 (0.25, 119.64), p=0.28  GNS: 1.65 (0.41, 6.71), p=0.48 |
| Unbooked mother | **Bhakri 2017**  8.12 (4.27, 15.45), p<0.0001 | - |

Unless otherwise specified, the values are unadjusted estimates. 95% CI are provided in brackets. aOR: Adjusted odds ratio

**Table S3B: Significant and non-significant factors from studies not included in meta-analysis for Ventilator-Associated Pneumonia (VAP) among neonates**

| **Risk factor** | **Studies- significant** | **Studies- non-significant** |
| --- | --- | --- |
| **NEONATAL** | |  |
| Sex | - | **Tripathi 2009**  not significant on multivariate analysis |
| Birth weight | **Tripathi 2009**  <VLBW: aOR: 3.88 (1.05,14.34), p=0.04 | - |
| SGA | - | **Tripathi 2009**  1.14 (0.19, 6.61) p=0.88 |
| Duration of MV | **Tripathi 2009**  aOR: 1.10 (1.02,1.21), p=0.02 | **Vijayakanthi 2015** |
| Resuscitation at birth | - | **Tripathi 2009**  1.69 (0.71,4.04), p=0.24 |
| Duration of hospitalization | **Tripathi 2009**  > 7days: 11.6 (3.39,40.22), p=0.0001  Mean (SD) VAP: 32.7 (34.7), No-VAP: 19.7 (23.9), p=0.003 | **Vijayakanthi 2015**  P=not significant |
| Reintubations | **Vijayakanthi 2015**  aOR: 34.3 (8.3, 142.4), p=0.001 | **Tripathi 2009**  aOR:0.75 (0.45, 1.23), p=0.25 |
| Level III stay | - | **Vijayakanthi 2015**  P=not significant |
| Unstable cardiopulmonary assessment at admission | - | **Vijayakanthi 2015**  aOR: 0.2 (0, 0.6), p=0.01 |
| Postnatal age | - | **Tripathi 2009**  0.71 (0.16, 3.20), p=0.66 |
| Respiratory distress at admission | - | **Vijayakanthi 2015**  P=not significant |
| **MATERNAL** | |  |
| Gestational age< 37 weeks | - | **Tripathi 2009**  aOR:1.16 (0.74,6.21), p=0.16 |
| PROM | - | **Tripathi 2009**  0.47 (0.19,1.12) |
| Place of delivery | - | **Vijayakanthi 2015**  P=not significant |

**Table S3C: Significant and non-significant factors from studies not included in meta-analysis for neonatal meningitis**

| **Risk factor** | **Studies- significant** | **Studies- non-significant** |
| --- | --- | --- |
| **NEONATAL** | |  |
| Birth weight | **DeNIS 2016a,b**  1.77 (1.29, 2.4), p=0.0003 | - |
| **MATERNAL** | |  |
| Gestational age | **DeNIS 2016a,b**  1.92 (1.45, 2.55), p<0.0001 | - |

**Table S3D: Significant and non-significant factors from studies not included in meta-analysis based on timing of onset of neonatal sepsis**

| **Risk factor** | **Studies- significant** | **Studies- Non-significant** |
| --- | --- | --- |
| **NEONATAL: early-onset sepsis** | | |
| Sex | **Dutta 2010**  aOR: 2.7 (1.55, 4.7) p=<.0001; score: 3* | - |
| Low birth weight | **Dutta 2010**  aOR: 2.79 (1.54, 5.09), p=0.001; score: 3* | **Santhanam 2017**  1.16 (0.48,2.8), p=0.77  1.78 (0.9, 3.19) |
| SGA | - | **Dutta 2010**  1.7 (0.9,2.8), p=0.05 |
| Resuscitation at birth | **Santhanam 2017**  2.58 (1.12, 5.90) |  |
| Major malformations | **DeNIS 2016a**  aOR: 1.55 (1.27,1.88) | - |
| APGAR score ≤4 at 5 minutes | - | **Dutta 2009**  1.2 (0.3,4.3), p=0.67 |
| **Late-onset sepsis** | | |
| Birth weight | - | **DeNIS 2016a**  aOR= 0.9 (0.9,1.0) |
| Duration of MV | **DeNIS 2016a**  <48 hours: aOR: 5.5 (3.4, 8.9)  >48 hours: aOR: 27 (10.5, 69.2) | - |
| IV fluids | **DeNIS 2016a**  <48 hours: aOR: 2.5 (1.8,3.6)  >48 hours: aOR: 11.6 (9.1,14.9) | - |
| **MATERNAL: Early-onset sepsis** | | |
| Gestational age | **DeNIS 2016a**  aOR: 0.8 (0.78,0.81)  **Dutta 2010**  <30 weeks: aOR:1.99 (1.12,3.53), p=0.02, score 2*  **Santhanam 2017**  <37 weeks: (pre-IAP) 2.86 (1.10,7.42), p=0.03  (Post-IAP) 0.35 (0.17, 0.73), p= 0.005 | - |
| Mode of delivery Caesarian | **DeNIS 2016a**  aOR: 0.72 (0.55,0.94)  **Santhanam 2017**  (post-IAP): 1.97 (1.15, 3.37), p=0.01 | **Santhanam 2017**  (pre-IAP): aOR: 1.43 (0.55, 3.71) |
| Primigravida | - | **Santhanam 2017**  (pre-IAP) 1.38 (0.71,2.68), p=0.34  (post-IAP) 1.47 (0.87,2.5), p=0.15 |
| Prolonged ROM  >18 hours  >24 hours | **DeNIS 2016a**  PROM>18 hrs: aOR: 1.91 (1.09,1.63)  **Santhanam 2017**  (pre-IAP): 5.29 (1.88,14.84), p=0.002  **Santhanam 2017**  (pre-IAP) aOR: 5.09 (1.44, 17.93) | **Dutta 2010**  PROM>18 hrs: 1.52 (0.8,2.8), p=0.16  **Santhanam 2017**  (post-IAP): 2.197 (0.4, 9.7)  **Santhanam 2017**  (post-IAP): 1 (0.21,4.81) |
| Spontaneous premature onset of labour | - | **Dutta 2010**  1.1 (0.7,1.7), p=0.83 |
| Preterm PROM | - | **Dutta 2010**  1.5 (0.9, 2.5), p=0.13 |
| Prelabour ROM | - | **Santhanam 2017**  (pre-IAP) 1.27 (0.57, 2.79), p=0.56  (post-IAP) 1.43 (0.69, 2.98), p=0.96 |
| Prolonged labour | - | **Dutta 2010 (**>18hrs)  0.62 (0.2,1.7), p=0.32 |
| multiple PV examination | **Dutta 2010** (≥3 in labour):  aOR:9.52 (2.97,30.53), p<0.0001; score:6*  **Santhanam 2017 (**>3 after ROM):  (pre-IAP) aOR: 11.22 (3.38, 37.22)  (post-IAP) aOR: 8.57 (3.10, 23.6) | - |
| Chorioamnionitis  FSL | **Dutta 2010 (**Chorioamnionitis)  aOR: 8.84 (1.81,43.17), p=0.007; score:6* | **Dutta 2010** FSL  0 (0, 24.96), p=0.73  **Santhanam 2017** - FSL zero events in cases (pre-IAP) and controls (post-IAP) |
| Meconium-stained amniotic fluid | **DeNIS 2016a**  aOR: 1.8 (1.43, 2.27)  **Santhanam 2017**  (post-IAP) aOR: 2.52 (1.18, 5.37) | **Santhanam 2017**  pre-IAP: 1.29 (0.57,2.92), p=0.55 |
| Maternal fever | **Santhanam 2017** (Peripartum fever)  (post-IAP) aOR: 3.54 (1.30, 9.67)  UTI: (post-IAP) aOR: 2.88 (1.08, 7.63) | **Dutta 2010** Intrapartum maternal fever: zero events in cases  **Santhanam 2017** (Peripartum fever)  (pre-IAP) aOR: 2.23 (0.57, 5.73)  UTI (pre-IAP) zero events in controls |
| IAP | **Dutta 2010**  aOR:2.07 (1.04, 4.11), p=0.04; score :2* | - |
| Number of antenatal care visits | **DeNIS 2016a**  >3 aOR: 1.34 (1.09,1.63) | - |
| **Late-onset sepsis** | | |
| Gestational age | - | **DeNIS 2016a**  aOR: 0.9 (0.8,1) |

Probability of developing EOS: *Score 3 to 4: 6.3 (1.3, 17.2), LR: 0.41 (0.13, 1.17); Score 2: 2.3 (0.3, 8.1), LR: 0.36 (0.51, 0.81); Score 5 to 6: 10.1 (6.6, 14.6), LR: 0.68 (0.47, 0.95)
