# Supplementary material for: Risk factors of neonatal sepsis in India: A systematic review and meta-analysis
Source: PLoS One. 2019 Apr 25;14(4):e0215683. doi: 10.1371/journal.pone.0215683 (PMC6483350; doi:10.1371/journal.pone.0215683)
Supplement: S4 Table — (DOCX) [file pone.0215683.s007.docx]

# **S4 Table**

# **Quality assessment of included studies**

# **Table S4A: Quality assessment of case-control studies (Newcastle Ottawa Scale)**

| **Study ID** | **Bhakri2017** | **Bhargava2017** | **Chaurasia2017** | **Das2016** | **Prashant2013** | **Santhanam2017** |
| --- | --- | --- | --- | --- | --- | --- |
| Selection | *** | ** | *** | ** | *** | ** |
| Comparability | - | ** | ** | * | - | * |
| Exposure | *** | *** | *** | *** | *** | *** |
| Quality | Fair | Good | Good | Fair | Fair | Fair |

# **Table S4B: Quality assessment of cohort studies (Newcastle Ottawa Scale)**

| **Study ID** | **Dutta2010** | **DeNISa,b2016** | **Tripathi2010** | **Vijayakanthi2015** |
| --- | --- | --- | --- | --- |
| Selection | *** | *** | *** | ** |
| Comparability | ** | ** | ** | ** |
| Outcome | *** | *** | ** | * |
| Quality | Good | Good | Good | Fair |

# **Table S4C: Quality assessment of cross-sectional studies (adapted Newcastle Ottawa Scale)**

| **Study ID** | **Pradhan2016** | **Soni2013** | **Sundaram2009** | **Tapader2014** | **Verma2015** |
| --- | --- | --- | --- | --- | --- |
| Selection | * | ** | ** | * | ** |
| Confounder | - | - | - | - | - |
| Outcome | * | * | * | * | * |
| Quality | Poor | Poor | Poor | Poor | Poor |

Notes:

Newcastle Ottawa Scale:

- Good, fair and poor quality were assigned based on conversion criteria used in Penson et al., 2012^[[1]](#footnote-1)^. The rationale for criteria were adapted from NOS quality form^1^ for cohort study used in Penson et al., 2012.

# **Table S4D: Quality assessment of observational cohort and cross-sectional studies (NHLBI)**

| **Question** | **Dutta 2010** | **DeNISa,b 2016** | **Pradhan 2016** | **Soni 2013** | **Sundaram 2009** | **Tapader 2014** | **Tripathi 2010** | **Vijayakanthi 2015** | **Verma 2015** |
| --- | --- | --- | --- | --- | --- | --- | --- | --- | --- |
| 1. Was the research question or objective in this paper clearly stated? | Y | Y | Y | Y | Y | Y | Y | Y | Y |
| 2. Was the study population clearly specified and defined? | Y | Y | CD | Y | Y | Y | Y | Y | Y |
| 3. Was the participation rate of eligible persons at least 50%? | Y | Y | Y | NR | Y | NR | Y | NR | Y |
| 4. Were all the subjects selected or recruited from the same or similar populations (including the same time period)? | CD | Y | CD | CD | Y | N | Y | Y | Y |
| Were inclusion and exclusion criteria for being in the study prespecified and applied uniformly to all participants? | Y | Y | Y | Y | Y | Y | Y | NR | Y |
| 5. Was a sample size justification, power description, or variance and effect estimates provided? | Y | NA | N | NA | NA | N | NA | NA | NA |
| 6. For the analyses in this paper, were the exposure(s) of interest measured prior to the outcome(s) being measured? | Y | Y | Y | Y | Y | CD | Y | CD | Y |
| 7. Was the timeframe sufficient so that one could reasonably expect to see an association between exposure and outcome if it existed? | Y | Y | Y | Y | Y | Y | Y | Y | Y |
| 8. For exposures that can vary in amount or level, did the study examine different levels of the exposure as related to the outcome (e.g., categories of exposure, or exposure measured as continuous variable)? | CD | Y | N | N | Y | N | CD | CD | N |
| 9. Were the exposure measures (independent variables) clearly defined, valid, reliable, and implemented consistently across all study participants? | Y | CD | NR | NR | Y | NR | CD | CD | CD |
| **Question** | **Dutta 2010** | **DeNISa,b 2016** | **Pradhan 2016** | **Soni 2013** | **Sundaram 2009** | **Tapader 2014** | **Tripathi 2010** | **Vijayakanthi 2015** | **Verma 2015** |
| 10. Was the exposure(s) assessed more than once over time? | CD | Y | CD | CD | NA | NR | Y | Y | NA |
| 11. Were the outcome measures (dependent variables) clearly defined, valid, reliable, and implemented consistently across all study participants? | Y | Y | Y | Y | Y | Y | Y | Y | Y |
| 12. Were the outcome assessors blinded to the exposure status of participants? | NR | NR | CD | NR | NR | NR | NR | NR | NR |
| 13. Was loss to follow-up after baseline 20% or less? | Y | Y | NA | NA | NA | CD | CD | CD | NA |
| 14. Were key potential confounding variables measured and adjusted statistically for their impact on the relationship between exposure(s) and outcome(s)? | Y | Y | N | N | N | N | Y | Y | N |
| Overall quality | Good | Good | Fair | Fair | Fair | Poor | Good | Poor | Fair |

Legend-Y: Yes, CD: Cannot Determine, N: No; NR: Not Reported; NA: Not Applicable

1. Penson DF, Krishnaswami S, Jules A, et al. Evaluation and Treatment of Cryptorchidism [Internet]. Rockville (MD): Agency for Healthcare Research and Quality (US); 2012 Dec. (Comparative Effectiveness Reviews, No. 88.) Appendix E, Quality of the Literature. Available from: https://www.ncbi.nlm.nih.gov/books/NBK115843/ [↑](#footnote-ref-1)
