# Supplementary material for: Risk factors of neonatal sepsis in India: A systematic review and meta-analysis
Source: PLoS One. 2019 Apr 25;14(4):e0215683. doi: 10.1371/journal.pone.0215683 (PMC6483350; doi:10.1371/journal.pone.0215683)

# **S1 Forest Plots**

# **Forest plots illustrating subgroup analysis by diagnostic criteria (Fig 12), by study design (Figs 13 and 14), and by study quality (Figs 14-18)**

# **S1A: Subgroup analysis by diagnostic criteria**

# Fig 12: Forest plot showing random-effects meta-analysis for male neonates with and without sepsis sub-grouped by sepsis diagnostic criteria (9 studies) [IV: Inverse Variance; CI: Confidence Interval]


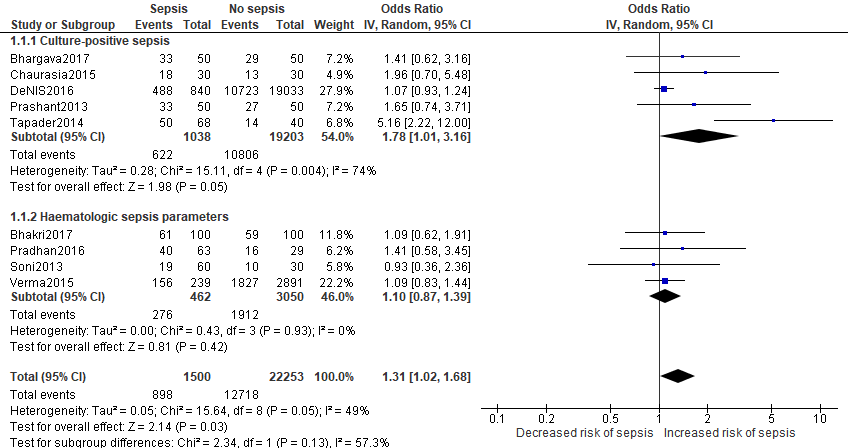


# **S1B: Subgroup analysis by study design**

# Fig 13: Forest plot showing random-effects meta-analysis for low birth weight (< 2500 grams) neonates with and without sepsis, sub-grouped by study design (4 studies) [IV: Inverse Variance; CI: Confidence Interval]


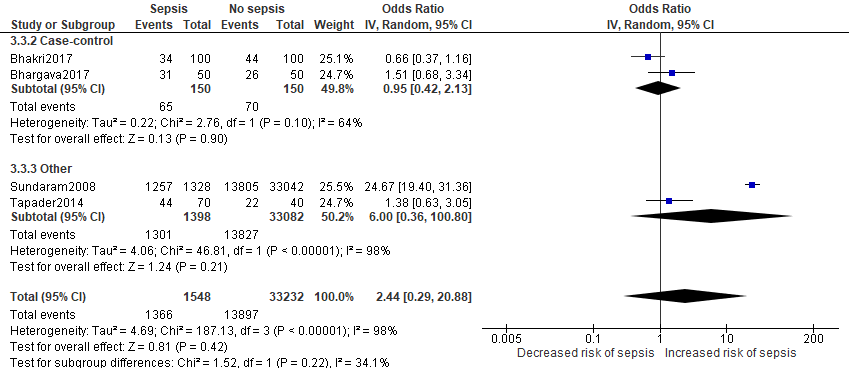


Fig 14: Forest plot showing random-effects meta-analysis of neonates, with and without sepsis, born to mothers delivering <37 weeks of gestation, sub-grouped by study design (6 studies) [IV: Inverse Variance; CI: Confidence Interval]


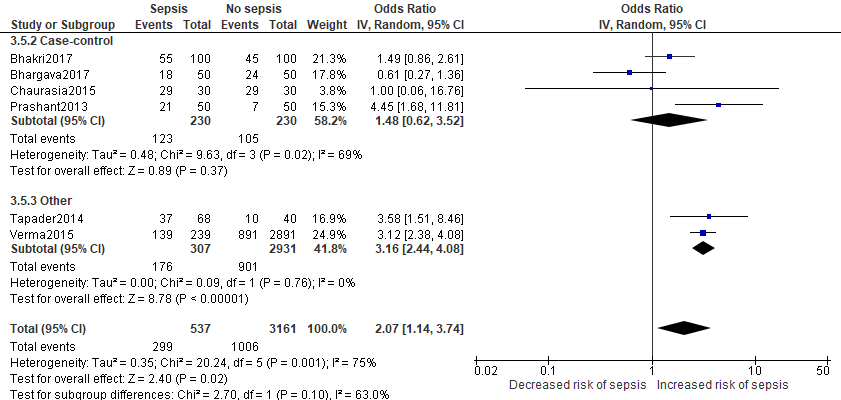


# **S1C: Subgroup analysis by study quality**

# Fig 15: Forest plot showing random-effects meta-analysis of male neonates, with and without sepsis, sub-grouped by study quality (9 studies) [IV: Inverse Variance; CI: Confidence Interval]


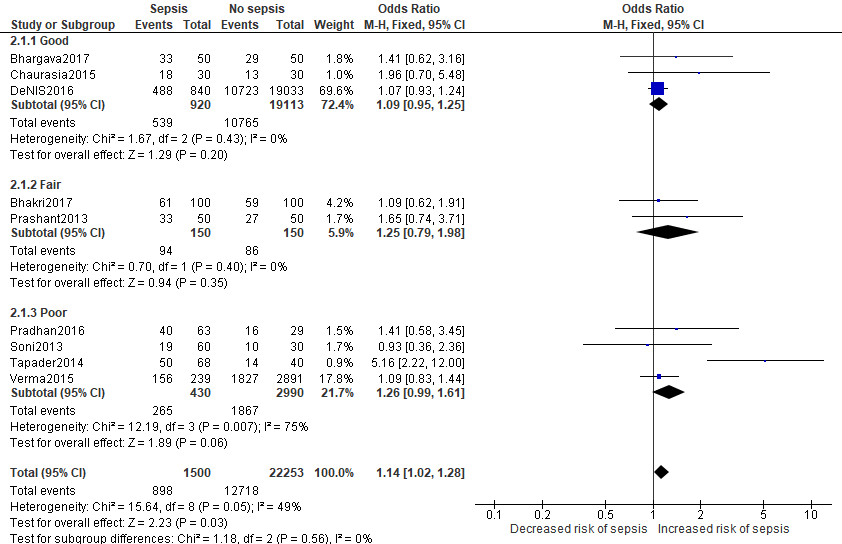


Fig 16: Forest plot showing random-effects meta-analysis for low birth weight (< 2500 grams) neonates with and without sepsis, sub-grouped by study quality (4 studies) [IV: Inverse Variance; CI: Confidence Interval]


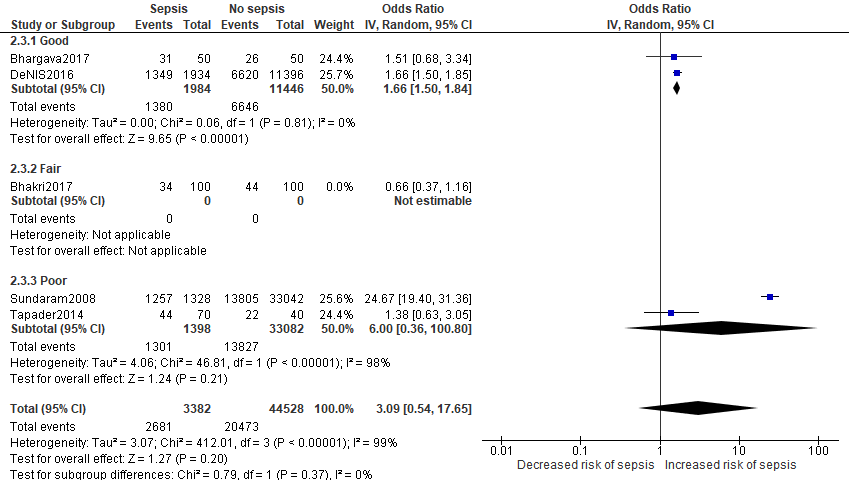


Fig 17: Forest plot showing random-effects meta-analysis of neonates, with and without sepsis, born to mothers delivering <37 weeks of gestation, sub-grouped by study quality (7 studies) [IV: Inverse Variance; CI: Confidence Interval]


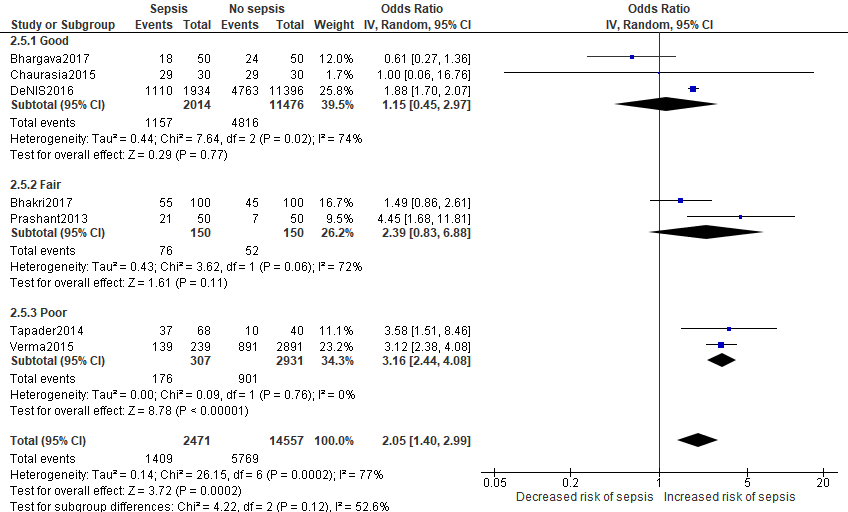


Fig 18: Forest plot showing random-effects meta-analysis of neonates, with and without sepsis, born to mothers who delivered vaginally, sub-grouped by study quality (4 studies) [IV: Inverse Variance; CI: Confidence Interval]


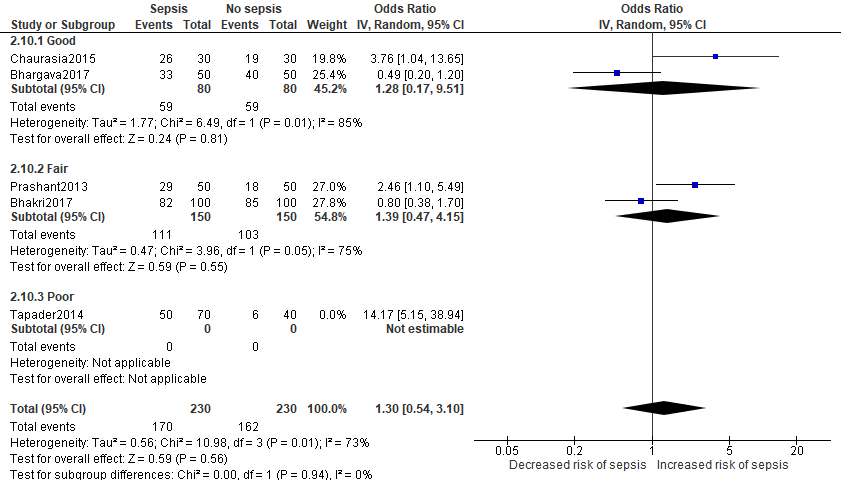

Supplement: S1 Forest Plots — (DOCX) [file pone.0215683.s008.docx]
