# Supplementary material for: Risk factors of neonatal sepsis in India: A systematic review and meta-analysis
Source: PLoS One. 2019 Apr 25;14(4):e0215683. doi: 10.1371/journal.pone.0215683 (PMC6483350; doi:10.1371/journal.pone.0215683)
Supplement: S1 Funnel Plots — (DOCX) [file pone.0215683.s009.docx]

**S1 Funnel Plots**

**Fig 19. Funnel plot illustrating publication bias assessment of male gender as a risk factor of neonatal sepsis**

**Fig 20. Funnel plot illustrating publication bias assessment for delivery <37 weeks of gestation as risk factor for neonatal sepsis.**

**Results of publication bias assessment^[[1]](#footnote-1)^**

| **Outcome** | **p-values** | |
| --- | --- | --- |
|  | **Egger’s test** | **Begg and Mazumdar’s test** |
| Male gender | 0.08 | 0.17 |
| Delivery <37 weeks of gestation | 0.83 | 0.76 |

1. Publication bias assessment (including funnel plot generation) was performed on Comprehensive Meta-Analysis V3 (Trial version): Borenstein M, Hedges L, Higgins J, Rothstein H. Biostat, Englewood, NJ 2013. [↑](#footnote-ref-1)
